# Recursive state and parameter estimation of COVID-19 circulating variants dynamics

**DOI:** 10.1038/s41598-022-18208-6

**Published:** 2022-09-23

**Authors:** Daniel Martins Silva, Argimiro Resende Secchi

**Affiliations:** Chemical Engineering Program/COPPE, Universidade Federal do Rio de Janeiro, Rio de Janeiro, 21941-942 Brazil

**Keywords:** Applied mathematics, Computational science, Epidemiology, Computational models

## Abstract

COVID-19 pandemic response with non-pharmaceutical interventions is an intrinsic control problem. Governments weigh social distancing policies to avoid overload in the health system without significant economic impact. The mutability of the SARS-CoV-2 virus, vaccination coverage, and mobility restriction measures change epidemic dynamics over time. A model-based control strategy requires reliable predictions to be efficient on a long-term basis. In this paper, a SEIR-based model is proposed considering dynamic feedback estimation. State and parameter estimations are performed on state estimators using augmented states. Three methods were implemented: constrained extended Kalman filter (CEKF), CEKF and smoother (CEKF & S), and moving horizon estimator (MHE). The parameters estimation was based on vaccine efficacy studies regarding transmissibility, severity of the disease, and lethality. Social distancing was assumed as a measured disturbance calculated using Google mobility data. Data from six federative units from Brazil were used to evaluate the proposed strategy. State and parameter estimations were performed from 1 October 2020 to 1 July 2021, during which Zeta and Gamma variants emerged. Simulation results showed that lethality increased between 11 and 30% for Zeta mutations and between 44 and 107% for Gamma mutations. In addition, transmissibility increased between 10 and 37% for the Zeta variant and between 43 and 119% for the Gamma variant. Furthermore, parameter estimation indicated temporal underreporting changes in hospitalized and deceased individuals. Overall, the estimation strategy showed to be suitable for dynamic feedback as simulation results presented an efficient detection and dynamic characterization of circulating variants.

## Introduction

The first official cases of COVID-19 were dated in December 2019 in Wuhan, China. Its spread worldwide in later months resulted in a pandemic classification from the World Health Organization (WHO) on 11 March 2020^1^. The first official case in Brazil was reported two weeks earlier, on 26 February 2020, from a man returning to São Paulo from Italy^2^. Social distancing presents effective mitigation over virus spread^3^; however, it generates negative impacts on the economy and on the mental health of the population^4^. Vaccination is a control action that progressively reduces virus transmissibility aiming for disease elimination defined by zero community infections^5^. Nonetheless, vaccination coverage is delimited by the vaccine acceptance rate, which makes its goal unfeasible even if vaccination provides 100% efficacy against transmission.

The SARS-CoV-2 virus is highly mutable, with thousands of variants documented since its origin in December 2019^6^. Mutations might change system dynamics; thus, model updating is required for reliable predictions. Genomic surveillance of SARS-CoV-2 virus in Brazil indicated four predominant circulating variants from February 2020 to July 2021. B.1.1.33 and B.1.1.28 were predominant from the pandemic beginning to September 2020; the Zeta variant (P.2), which originated in Rio de Janeiro, was predominant from October 2020 to February 2021; and the Gamma variant (P.1), which originated in Amazonas, was predominant from mid-February 2021 to July 2021^7^. In the pandemic modeling, the dynamics from each variant correspond to a set of parameters that must be estimated to ensure an accurate prediction over an extended period of analysis.

Modeling epidemiological evolution by a compartmental model is standard for control-oriented models since its simplicity suits real-time applications^8–12^. Optimality is usually defined to mitigate virus spread within health system capacity, while an input or manipulated variable is correlated to contagion rate. The input variable is discrete for a definition based on previously implemented government restrictive measures^8,10,11^; and continuous for a definition based on mobility data^12^ or possible government measures (e.g., complete lockdown and no countermeasures)^9^. Nonetheless, model parameters are not constrained to functions of the manipulated variables. Olivier et al.^8^ defined several compartmental model parameters as time-varying functions. Köhler et al.^9^ described hospitalized parameters as a function of the state variables. Morato et al.^10^ used a three-step parameter estimation of contagion, recovery, and mortality rates.

Mobility data is regarded generally in models focused on the forecast^13–19^. There are many available database, among which there are data related to local infection probability and restrictive government measures. For instance, SafeGraph details node-related information fit for a network model^13^. Facebook details geographic movement metrics suitable for spatial models^14,15^. Apple and Google detail location mobility trends correlated to government measures which are used coupled^16,17^ or standalone^18,19^. In addition, mobility dynamics might be identified and described on time-varying functions from previous government measures^20,21^.

Virus mutations and vaccination coverage affect model dynamics, adding uncertainties to the system. There are examples of recursive state and parameter estimation applications in the COVID-19 pandemic. Sun et al.^22^ estimated parameters at each discrete time with a grid search, while Menda et al.^23^ estimated them with a neural network. Liao et al.^24^ and Morato et al.^10^ estimated parameters with moving horizon estimation based on a least-square method followed by a regressive method; moreover, the latter^10^ proposed an additional moving average on the estimation structure. Tsay et al.^25^ estimated unmeasured states with an unscented Kalman filter. Zhu et al.^26^ estimated states and parameters into an augmented state with an extended Kalman filter (EKF). Song et al.^27^ estimated states with an EKF and parameters with a proposed strategy based on maximum likelihood. State and parameter estimations in the literature focused on overall system dynamics. The authors used estimation strategies to estimate unmeasured states, capture reinfection dynamics or adjust model parameters for more accurate estimations. Hence, virus mutations and vaccination dynamics have not been study objects with similar estimation strategies.

Transmissibility, severity of the disease, and lethality are three properties of interest for study in an epidemiological model. They are defined by the probability of an infected individual moving from one given compartment to another. Marziano et al.^28^ proposed an age-structured model to analyze the Italia epidemic evolution during its first wave for possible outcomes from easing restrictive measures. The transmissibility was a function of google mobility data, the probability of developing severe disease was a fitting parameter per age group, and the lethality was defined as a function of the latter and hospitalized data. Kemp et al.^21^ proposed a compartmental model with fitting parameters for each probability of split in the model configuration to analyze herd immunity in Austria, Luxemburg, and Sweden. The transmissibility was a function of mobility fitted for each previous government measure, while other parameters were fitted as constants for each wave of COVID-19 infection.

In this work, we propose a comprehensive compartmental model for detecting epidemiological dynamics in terms of transmission, severity of the disease, and lethality equivalent to vaccine efficacy studies. Classical vaccination coverage modeling through a SIR-based model supposes the vaccinated state is 100% immune to reinfection; however, recent literature contradicted this assumption^29^. The modeling through correlated vaccination parameters is an alternative formulation to comprehend the vaccine dynamics. Hence, it is suitable for analyzing vaccine efficacy or intervention measures. The proposed model considers a recursive estimation approach in which simplifying assumptions focuses on detecting the aforementioned dynamics with parameter estimation. Model accuracy is improved using temporal prevalence distributions from the seroprevalence survey EPICOVID19-BR in the first wave of the pandemic. The proposed estimation strategy identifies COVID-19 variant emergence and characterizes its dynamics on epidemiological evolution based on dynamic feedback, which is suitable for online applications.

First, we describe the proposed compartmental model assumptions and parameter identification of the first wave of the pandemic. Then, we describe the implemented state and parameter estimation strategies. Next, we show numerical results from simulations on several Brazilian federative units and analyze the estimated parameters. Finally, we make our conclusions and discuss possible future works.

## Mathematical model

Predicting the dynamics of an epidemiological evolution is of utmost importance to control its spread in a population. Modeling by a SIR-based model is conventional in control applications because of its simplicity and real-time applicability. The SARS-CoV-2 virus, however, presents high mutability, which affects model parameters over time. In addition, a relevant percentage of the population has been getting vaccinated in 2021, which also affects those parameters. Both uncertainty sources were irrelevant in the early stages of the COVID-19 pandemic, but their systematic increase make long-term forecasts unreliable. Hence, an accurate system estimate over an extended analysis period requires state feedback. In this work, the state feedback was done by simultaneous state and parameter estimation using an augmented vector.

In this section, a compartmental model is adapted to improve estimation performance considering data availability in Brazil. The model in Equation (1) was adapted from the SIDARTHE model proposed by Giordano et al.^30^ Hence, it assumes homogeneous states without age structure or the effect of vaccination coverage. The estimated parameters $\alpha _0$, $x_c$, and $x_m$ related to transmissibility, severity of the disease, and lethality, respectively, are described later in this section. These parameters correlate with the dynamics analyzed in COVID-19 vaccine effectiveness studies^31–34^. We have selected a federative unit per Brazilian region to evaluate epidemic progression countrywide, but the southeast region, the most populated one, is an exception with two units. Amazonas (AM) was chosen for the north region; Mato Grosso do Sul (MS) for the central-west; Rio Grande do Norte (RN) for the northeast; Rio Grande do Sul (RS) for the south; Rio de Janeiro (RJ) and São Paulo (SP) for the southeast. The total population $N_i$ from each federative unit *i* consists of the following compartments:

- Susceptible (*S*): individuals prone to infection;
- Exposed (*E*): individuals infected in the incubation period, while they are not infectious;
- Infected (*I*): undetected asymptomatic individuals;
- Quarantined (*Q*): detected asymptomatic individuals who self-quarantine after detecting the disease;
- Ailed (*A*): undetected symptomatic individuals;
- Recognized (*R*): detected symptomatic individuals who self-quarantine after detecting the disease;
- Threatened (*T*): individuals hospitalized in nursery or intensive care units (ICU);
- Healed detected ($H_d$): detected individuals cured without treatment;
- Deceased (*D*): individuals deceased due to the disease;
- Healed with treatment ($H_t$): individuals cured after a hospitalization period;
- Healed undetected ($H_u$): individuals cured of the disease without being detected.

1a $$\begin{aligned} \frac{d S}{dt}&= - \nu S \end{aligned}$$

1b $$\begin{aligned} \frac{d E}{dt}&= \nu S - \rho E \end{aligned}$$

1c $$\begin{aligned} \frac{d I}{dt}&= p \rho E - (\lambda +\varepsilon ) I \end{aligned}$$

1d $$\begin{aligned} \frac{d Q}{dt}&= \varepsilon I - \lambda _{d} Q \end{aligned}$$

1e $$\begin{aligned} \frac{d A}{dt}&= (1-p) \rho E - (\theta +\mu +\kappa ) A \end{aligned}$$

1f $$\begin{aligned} \frac{d R}{dt}&= \theta A - (\mu _{d}+\kappa _{d}) R \end{aligned}$$

1g $$\begin{aligned} \frac{d T}{dt}&= \mu A +\mu _{d} R - (\sigma +\tau ) T \end{aligned}$$

1h $$\begin{aligned} \frac{d H_d}{dt}&= \lambda _{d} Q + \kappa _{d} R \end{aligned}$$

1i $$\begin{aligned} \frac{d D}{dt}&= \tau T \end{aligned}$$

1j $$\begin{aligned} \frac{d H_{t}}{dt}&= \sigma T \end{aligned}$$

1k $$\begin{aligned} \frac{d H_{u}}{dt}&= \lambda I + \kappa A \end{aligned}$$

where all states are fractions of a total population $N_i$, informed by the Ministry of Health of Brazil^2^. $\nu$ is the infection rate, $\rho$ is the incubation rate, and *p* is the fraction of infected individuals who remain asymptomatic. $\varepsilon$ and $\theta$ are the detection rates of *I* and *A*, respectively. $\lambda$, $\lambda _{d}$, $\kappa$, $\kappa _{d}$, and $\sigma$ are the recovery rates of *I*, *Q*, *A*, *R*, and *T*, respectively. $\tau$ is the mortality rate, whereas $\mu$ and $\mu _{d}$ are severe illness rates of *A* and *R*, respectively. Fig. 1 shows a scheme of the state transitions.

**Figure 1:**
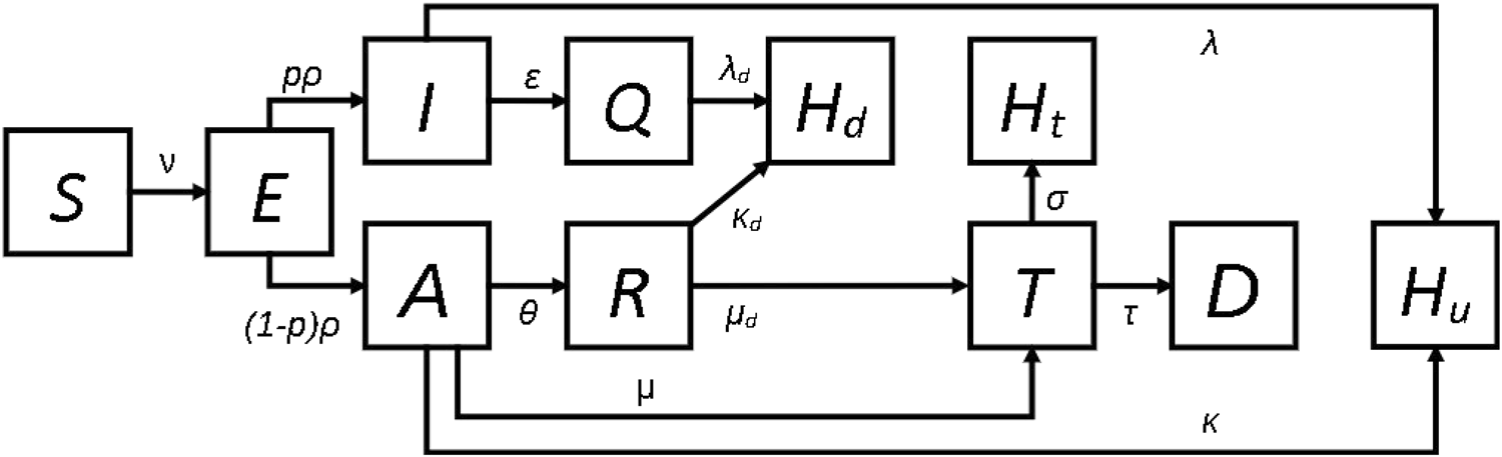
Schematic diagram of the proposed compartmental model.

The analysis of several cases studies within a larger region provides spatial dynamics concerning virus spread. The chosen federative units for the study are known to be heterogeneous among each other^35–37^ since Brazil is a large country where there were several different outbreaks dates, local government policies, and population behavior. Brazilian spatial epidemic progression studies^35,36^ indicated multiple initial outbreaks spread progressively to neighboring territories. The numerous government policies and population behavior are pointed out by the higher variance of the first wave duration of Brazilian states when compared to the United States and India variances^37^.

The healed compartment from the Giordano et al. model^30^ is subdivided into three compartments: $H_{d}$, $H_{u}$, and $H_{t}$. $H_{t}$ is a measurable state by the Brazilian severe acute respiratory syndrome (SARS) database^38,39^. $H_{d}$ is an unmeasured state because the Brazilian SARS database accounts only for the hospitalized individuals, and the Ministry of Health of Brazil only provides recovered estimate countrywide. However, the cumulative confirmed cases provided by the latter are composed mainly of $H_{d}$ for any analysis post the first wave. $H_{u}$ is an unmeasured state containing most post-infection individuals for all studied federative units. The closed system assumption of the compartmental model leads to the following constraint: $S + E + I + Q + A + R + T + H_d + D + H_t + H_u = 1$; thus, we substituted Equation (1k) by Equation (2).

2 $$\begin{aligned} H_{u} = 1 - S - E - I - Q - A - R - T - H_d - D - H_t \end{aligned}$$

The additional state *E* corresponds to a natural time delay of the system, which is usual in control-oriented models^8,11^ and forecasts to a lesser extent^20,40,41^. Presymptomatic infected individuals are within this state as $(1-p)E$, but their infection rate is assumed insignificant to simplify parameter estimation.

Reinfections play a significant role in the resurgence of COVID-19 infection waves since new lineage might evade immunity from previous infections^42^. Gamma^43^ and Delta^44^ mutations allow them to infect individuals recovered from other variants. Hence, reinfections from natural immunity decrease are assumed negligible to the emergence of another variant. The latter, however, can not be forecasted as they happen in occasional events. Hence, *S* in the model Equation (1) is unconnected with healed compartments $H_u$, $H_t$, and $H_d$, and the reinfection dynamics are assumed to be comprised in the state estimation.

The infection rate is simplified into a single parameter to guarantee observability. Hence, infections caused by presymptomatic and detected infected are assumed to be insignificant compared to infections caused by undetected infected. In addition, the same infection rate is applied to symptomatic and asymptomatic, although the first is acknowledged as more infectious^45^.

3 $$\begin{aligned} \nu = \alpha (I + A) \end{aligned}$$

where $\alpha$ is the contagion rate, consisting of the probability that a susceptible individual contracts the disease from possible contact with an infectious individual. It is a function of non-pharmaceutical interventions (NPI), vaccination coverage, and circulating variants. NPI and vaccination mitigate virus spread in the short-term, while virus mutations might affect its transmissibility, as happened for the Gamma^43^ variant.

NPI dynamics are inserted into a compartmental model by time-varying functions^20^, independent variables^9,11,12^, or time-varying parameters estimated over time^24,25^. We focused on these last two as they are better suited to a control-oriented model. First, we separated social distancing from other NPI by defining $\alpha$ according to Equation (4).

4 $$\begin{aligned} \alpha = \alpha _{0} (1 - u) \end{aligned}$$

where $u \in [0,1]$ is the manipulated variable related to social distancing and $\alpha _{0}$ is the estimated contagion rate. The linearity applied over $\alpha$ and *u* in Equation (4) gets the correct direction between the contagion rate and social distancing. NPI unrelated to social distancing (e.g., mass gathering restrictions and mask requirements) are comprised in $\alpha _{0}$.

Social distancing is measurable by Google mobility data as percentage changes concerning a baseline defined from data sets before the COVID-19 outbreak^46^. Google mobility data are divided into six categories: recreation, essentials, parks, transit, workplace, and resident. A linear combination among the two most independent categories is used to define *u*. The similarity was measured by a zero-lag cross-correlation matrix through data from all federative units studied between February 2020 and July 2021. The normalized cross-correlation, whose results are presented in Supplementary Table S1, was calculated using the xcorr function from MATLAB. The absolute difference from zero characterizes the similarity between two signals, where independence is defined. The essentials signal had a cross-correlation closer to zero for all categories except itself; however, it is a monthly periodic signal while the others are weekly reported. Hence, the cross-correlation closest to zero, disregarding essentials, is related to parks and workplace; thus, they were selected to define *u*. Additionally, *u* was limited in the range [0,1], assuming each mobility category has lower and upper bounds on -100% and 100%, respectively. A weighted sum to assimilate location-dependent correlations concerning each mobility category was used to evaluate *u* according to Equation (5).

5 $$\begin{aligned} \begin{aligned} u(t)&=\dfrac{-w_u\left\langle Parks(t)\right\rangle -(1-w_u)\left\langle Workplace(t)\right\rangle -(w_u Parks_{min}+(1-w_u) Workplace_{min})}{w_u (Parks_{max}-Parks_{min})+(1-w_u) (Workplace_{max}-Workplace_{min})}\\&= \dfrac{-w_u \left\langle Parks(t)\right\rangle -(1-w_u)\left\langle Workplace(t)\right\rangle -(w_u (-100)-(1-w_u)(-100))}{w_u(100-(-100))+(1-w_u)(100-(-100))}\\&= \dfrac{-w_u \left\langle Parks(t)\right\rangle -(1-w_u) \left\langle Workplace(t)\right\rangle +100}{200} \end{aligned} \end{aligned}$$

where $\left\langle Parks(t)\right\rangle$ and $\left\langle Workplace(t)\right\rangle$ are weekly moving averages of parks and workplace, respectively, and $w_u$ is the relative weight concerning parks mobility, which is an additional parameter to be estimated in the model identification.

The fraction of individuals who do not experience symptoms is defined as $p \in [0.15,0.7]$ according to the U.S. Centers for Disease Control and Prevention (CDC)^47^. Virus mutations, testing policies, and different age distribution explain the broad range. The parameter *p* is not estimated over time because it is not observable from available data since there is no classification of symptomatic and asymptomatic. Hence, we defined $p=0.5$ as an intermediate value whose error is mitigated by the state estimator with estimations of *I* and *A*. The vaccine efficacy against infection is correlated to both $\alpha _{0}$ and *p* since it only measures symptomatic cases, according to U.S. Food and Drug Administration (FDA).

Following the vaccine efficacy against severe and mild diseases, a parameter $x_c$ is defined as the fraction of symptomatic individuals who develop severe or mild symptoms. Assuming that severe and mild illnesses imply hospitalization, thus $x_c$ is the fraction of individuals moving from *A* and *R* to *T*. Summing up Equations (1e) and (1f):

$$\begin{aligned} \frac{d(A+R)}{dt}= (1-p) \rho E - (\mu +\kappa ) A - (\mu _{d}+\kappa _{d}) R \end{aligned}$$

which is simplified by assuming $\mu \approx \mu _{d}$ and $\kappa \approx \kappa _{d}$ to:

$$\begin{aligned} \frac{d(A+R)}{dt}= (1-p) \rho E - (\mu +\kappa ) (A + R) \end{aligned}$$

Thus:

$$\begin{aligned} x_{c} = \frac{\mu }{\mu + \kappa } \end{aligned}$$

Let us rewrite $\mu$ and $\kappa$ as a probability function of the symptomatic individual to follow their ways, then:

6 $$\begin{aligned} x_{c} = \dfrac{(1-x_k-x_{\theta }) {\tilde{\mu }}}{(1-x_k-x_{\theta }) {\tilde{\mu }} + x_k {\tilde{\kappa }}} \end{aligned}$$

where $x_k$ and $x_{\theta }$ are the probabilities of an individual in *A* to recover or to get detected, respectively. The average rates of severe illness ${\tilde{\mu }}$ and symptomatic recovery ${\tilde{\kappa }}$ correspond to properties studied in the literature. In this work, we defined ${\tilde{\mu }} = 1/5\ \text {d}^{-1}$ and $\rho = 1/5.2\ \text {d}^{-1}$ from CDC^47^, and ${\tilde{\kappa }}$ was based on a study of the detection window and test sensitivity of IgG/IgM tests^48^. The testing rate is a local and time-dependent property that affects both the probabilities $x_k$ and $x_{\theta }$. Let us define the correlated parameter $x_s$ as the fraction of recovered undetected individuals. We have from Equation (1e):

7 $$\begin{aligned} x_{s} = \dfrac{\kappa }{\mu + \theta + \kappa } = \dfrac{ x_k {\tilde{\kappa }}}{(1-x_k-x_{\theta }) {\tilde{\mu }} + x_{\theta } {\tilde{\theta }} + x_k {\tilde{\kappa }}} \end{aligned}$$

Rewriting Equation (7) for $x_k$:

$$\begin{aligned} x_{k} = \dfrac{x_s x_{\theta } {\tilde{\theta }} + (1-x_{\theta }) x_s {\tilde{\mu }} }{(1-x_s) {\tilde{\kappa }} + x_s {\tilde{\mu }}} \end{aligned}$$

and substituting it in Equation (6) rewritten for $x_{\theta }$:

8 $$\begin{aligned} x_{\theta } = \dfrac{(1-x_c-x_s) {\tilde{\kappa }} {\tilde{\mu }}}{(1-x_c-x_s) {\tilde{\kappa }} {\tilde{\mu }} + (1-x_c) x_s {\tilde{\theta }} {\tilde{\mu }} + x_c x_s {\tilde{\theta }} {\tilde{\kappa }}} \end{aligned}$$

Considering that $x_{c}$ and $x_{s}$ represent fractions of the symptomatic infected, then $x_{s}+x_c \in [0,1]$. Locations with a steadier testing policy could estimate $x_s$ as a constant. However, rapid tests and RT-PCR were not available in public health services in the early stages of the COVID-19 pandemic in Brazil. Defining $x_s$ as a logistic equation in the function of time according to:

9 $$\begin{aligned} x_{s} = a_{x_{s}} \left( 1-\frac{a_{\zeta }}{1+\exp {\left( -b_{\zeta } (t - c_{\zeta })\right) }}\right) \end{aligned}$$

where $a_{x_{s}}$, $a_{\zeta }$, $b_{\zeta }$, and $c_{\zeta }$ are identified model parameters. The definition of Equation (9) is based on heuristics that $x_s$ is initially high and decreases progressively to a steady state following test availability to the population. These model parameters also comprises uncertainties regarding test policy.

Analogous to $x_s$, we define the fraction of recovered undetected asymptomatic individuals $x_{a}$ from Equation (1c) as:

$$\begin{aligned} x_{a} = \frac{\lambda }{\lambda +\varepsilon } = \frac{x_{id} {\tilde{\lambda }}}{x_{id} {\tilde{\lambda }} + (1- x_{id}) {\tilde{\varepsilon }}} \leftrightarrow x_{id} = \frac{x_{a} {\tilde{\varepsilon }}}{{\tilde{\lambda }} + x_{a} ({\tilde{\varepsilon }} - {\tilde{\lambda }})} \end{aligned}$$

where $x_{id}$ is the probability of detecting the disease in an asymptomatic individual. Defining $x_a$ similarly to $x_s$, we have:

10 $$\begin{aligned} x_{a} = a_{x_{a}} \left( 1-\frac{a_{\zeta }}{1+\exp {\left( -b_{\zeta } (t - c_{\zeta })\right) }}\right) \end{aligned}$$

where $a_{x_{a}}$ is an additional identified model parameter. Equations (9) and (10) have linear dependence between $x_{s}$ and $x_{a}$ to avoid overfitting of an excessive number of model parameters. The ratio $a_{x_{a}}/a_{x_{s}}$ comprises the effect of the viral load on the test sensitivity and the test probability between symptomatic and asymptomatic infected individuals. Related uncertainties are assumed to be mitigated by state estimation among the states *I*, *Q*, *A*, and *R*.

Finally, we define a parameter $x_m$ analogous to vaccine efficacy against lethality as the fraction of threatened individuals who decease. We define it from Equation (1g) as:

11 $$x_{m} = \frac{{\mathbf{\tau }}}{{\sigma + {\mathbf{\tau }}}}$$

Rewriting Equation (11) as a function of a death probability $x_e$:

$$\begin{aligned} x_{m} = \frac{x_e {\tilde{\mathbf{\tau } }}}{(1-x_e){\tilde{\mathbf{\sigma} }} + x_e {\tilde{\mathbf{\tau } }}} \end{aligned}$$

and isolating $x_e$ give us:

12 $$\begin{aligned} x_{e} = \frac{x_m {\tilde{\mathbf{\sigma} }}}{(1-x_m){\tilde{\mathbf{\sigma} }} + x_m {\tilde{\sigma }}} \end{aligned}$$

where ${\tilde{\sigma }}$ is the average recovery rate from hospitalization and ${\tilde{\tau }}$ is the average mortality rate. These parameters depend on healthcare demand, medical resources, notification delay, virus mutations, vaccine coverage, and testing policy. Nonetheless, they are simplified as constants to allow future estimations since, by assumption, uncertainties are mitigated by the state and parameter estimation.

The definition of parameters equivalent to vaccine efficacy against transmissibility, severity of the disease, and lethality as functions of state transition rates give comprehensive information about the virus spreading dynamics. The model uncertainties are outweighed by better parameter estimations by considering a fewer number of estimated parameters. The definition of $x_c$ and $x_m$ yields additional flexibility in the model formulation. Minor changes applied over $\alpha _0$, $x_m$, and $x_c$ can express specific vaccine dynamics on the model. Hence, their definition comprehends an alternative implementation of vaccination in compartmental modeling.

The Ministry of Health of Brazil^2^ provides accumulated data on confirmed cases, deceased, and their respective incidences for each federative unit and county. The Brazilian SARS database^38,39^ provides clinical data from patients with a severe acute respiratory syndrome which comprehend confirmed and suspected cases of COVID-19 and other diseases. It notifies the period of hospitalization, evolution date, the confirmation status of COVID-19, among other information. Summing up all confirmed COVID-19 patients per each federative unit *i* gives observability on $T_i$ and $H_{t,i}$. In addition, overall means of hospitalized evolution between April 2021 and July 2021 were used to define ${\tilde{\sigma }}$ and ${\tilde{\tau }}$. Both databases are daily measured; hence sampling time $T_s = 1$ d. Average testing rates ${\tilde{\varepsilon }}$ and ${\tilde{\theta }}$ are location-dependent; however, we assumed that correlated uncertainties are comprehended in $a_{\zeta ,i}$, $b_{\zeta ,i}$ and $c_{\zeta ,i}$. Hence, we defined ${\tilde{\varepsilon }} = {\tilde{\theta }}$ = $1\ \text {d}^{-1}$ to suit sampling time. In summary, the monitored variable $\mathbf {y_i}$ is defined as:

13 $$\begin{aligned} \mathbf {y_i}(k) = \mathbf {h}(\mathbf {x}_{\mathbf {i}}) = \begin{bmatrix} Q_i(k)+R_i(k)+T_i(k)+H_{d,i}(k) + D_i(k) + H_{t,i}(k)\\ D_i(k)\\ T_i(k)\\ H_{t,i} (k)\end{bmatrix} \end{aligned}$$

where $$\mathbf {x_i} = \left[ S_i\ E_i\ I_i\ Q_i\ A_i\ R_i\ T_i\ H_{d,i}\ D_i\ H_{t,i} \right] ^T$$.

EPICOVID19-BR provides additional data over temporal distributions in Brazil. It surveyed COVID-19 prevalence in cities from all regions on different timelines^49,50^. Let us consider the prevalence estimations from federative units given by Marra and Quartin^51^ based on three phases of EPICOVID19-BR. Furthermore, if we assume ${\tilde{\lambda }}={\tilde{\kappa }}=1/15\ \text {d}^{-1}$, then we can correlate states $H_{d,i}$ and $H_{u,i}$ with test sensitivity. EPICOVID19-BR did not test hospitalized patients^49^ and used an IgM and IgG antibody test more sensitive 15 days after the appearance of symptoms^48^. Hence, the state transition model $\mathbf {f}(\mathbf {x_i},u_i)$ for each federative unit *i* is defined in Equation (14).

14 $$\begin{aligned} \begin{aligned} \frac{d S_i}{dt}&= - \nu _i S_i \\ \frac{d E_i}{dt}&= \nu _i S_i - \rho E_i \\ \frac{d I_i}{dt}&= p \rho E_i - (\lambda _i+\varepsilon _i) I_i \\ \frac{d Q_i}{dt}&= \varepsilon _i I_i - \lambda _i Q_i \\ \frac{d A_i}{dt}&= (1-p) \rho E_i - (\theta _i+\mu _i+\kappa _i) A_i \\ \frac{d R_i}{dt}&= \theta _i A_i - (\mu _i+\kappa _i) R_i \\ \frac{d T_i}{dt}&= \mu _i (A_i + R_i) - (\sigma _i+\tau _i) T_i\\ \frac{d H_{d,i}}{dt}&= \lambda _i Q_i + \kappa _i R_i \\ \frac{d D_i}{dt}&= \tau _i T_i\\ \frac{d H_{t,i}}{dt}&= \sigma _i T_i\\ H_{u,i}&= 1 - S_i - E_i - I_i - Q_i - A_i - R_i - T_i - H_{d,i} - D_i - H_{t,i}\\ \nu _i&= \alpha _{0,i} (1 - u_i) (I_i + A_i)\\ x_{a,i}&= a_{x_{a,i}} \left( 1-\frac{a_{\zeta ,i}}{1+\exp {\left( -b_{\zeta ,i} (t - c_{\zeta ,i})\right) }}\right) ,\ x_{s,i} = a_{x_{s,i}} \left( 1-\frac{a_{\zeta ,i}}{1+\exp {\left( -b_{\zeta ,i} (t - c_{\zeta ,i})\right) }}\right) ,\\ x_{id,i}&= \frac{x_{a,i} {\tilde{\varepsilon }}}{{\tilde{\lambda }} + x_{a,i} ({\tilde{\varepsilon }} - {\tilde{\lambda }})}\\ x_{k,i}&= \frac{x_{s,i} x_{\theta ,i} {\tilde{\theta }} + (1-x_{\theta ,i}) x_{s,i} {\tilde{\mu }} }{(1-x_{s,i}) {\tilde{\kappa }} + x_{s,i} {\tilde{\mu }}},\\ x_{\theta ,i}&= \frac{(1-x_{c,i}-x_{s,i}) {\tilde{\kappa }} {\tilde{\mu }}}{(1-x_{c,i}-x_{s,i}) {\tilde{\kappa }} {\tilde{\mu }} + (1-x_{c,i}) x_{s,i} {\tilde{\theta }} {\tilde{\mu }} + x_{c,i} x_{s,i} {\tilde{\theta }} {\tilde{\kappa }}},\ x_{e,i} = \frac{x_{m,i} {\tilde{\sigma }}_i}{(1-x_{m,i}){\tilde{\tau }}_i + x_{m,i} {\tilde{\sigma }}_i}\\ \lambda _i&= (1-x_{id,i}) {\tilde{\lambda }},\ \varepsilon _i = x_{id,i} {\tilde{\varepsilon }},\ \theta _i = x_{\theta ,i} {\tilde{\theta }},\ \kappa _i = x_{k,i} {\tilde{\kappa }},\ \mu _i = (1- x_{k,i} - x_{\theta ,i}) {\tilde{\mu }},\\ \sigma _i&= (1-x_{e,i}) {\tilde{\sigma }}_i,\ \tau _i = x_{e,i} {\tilde{\tau }}_i \end{aligned} \end{aligned}$$

Each federative unit *i* under study has prevalence distribution from EPICOVID19-BR formulated as Equation (15) for each phase $j\in \{1,2,3\}$, assuming a test sensitivity of 100% during $T_{p}$ days followed by a sudden decay to zero.

15 $$\begin{aligned} \begin{aligned} Pre_{i,j,min} \le \frac{1}{N_{total,j}} \sum _{k=0}^{N_{total,j}-1} \left( H_{all,i}(N_{ep,j}+k) - H_{all,i}(N_{ep,j}+k-T_{p}))\right) \le Pre_{i,j,max} \end{aligned} \end{aligned}$$

where prevalence bounds $Pre_{i,j,min}$ and $Pre_{i,j,max}$ can be found in Supplementary Table S2^51^, and $T_{p} = 50$ was the arbitrated value for the detection window. $t(N_{ep,1})=\text {14 May 2020}$, $t(N_{ep,2})=\text {4 June 2020}$, $t(N_{ep,3})=\text {21 June 2020}$ are initial dates from the first, second and third phases of EPICOVID19-BR, respectively, while $N_{total,1}=8$ and $N_{total,2}=N_{total,3}=4$ correspond to their respective duration in days, and $H_{all,i} = H_{u,i}+H_{d,i}+H_{t,i}$. In the early stages of the pandemic outbreak, recovered individuals are approximately null; thus, we defined $H_{d,i}(N_{ep,j}+k-T_{p})=H_{t,i}(N_{ep,j}+k-T_{p})=H_{u,i}(N_{ep,j}+k-T_{p})=0 \forall \left\{ N_{ep,j}+k<T_{p}| j \in \{1,2,3\} \right\}$.

Gene sequences reported in GISAID^7^ indicate Zeta variant appearance in mid-October 2020. Hence, the identification step is bounded at $t(N_f) = \text {1 October 2020}$ to guarantee the steady circulation of variants B.1.1.28 and B.1.1.33. The lower bound aims at an imported infection neglectful in the system when $\left[ I_i(t_{0,i})\ Q_i(t_{0,i})\ A_i(t_{0,i})\ R_i(t_{0,i})\ T_i(t_{0,i})\ H_{d,i}(t_{0,i})\ D_i(t_{0,i})\ H_{t,i}(t_{0,i})\ H_u(t_{0,i}) \right] ^T \approx {{\textbf {0}}}$. Therefore, only $S_i (t_{0,i})$ and $E_i (t_{0,i})$ were considered optimization variables for the model identification. Hospitalized individuals $T_i$ were used to define $\{N_{0,i}|t_{0,i} = t(N_{0,i})\}$ from the solution of a system composed by $T_i(N_{0,i})>0.00003$, $T_p-N_{ep,3}-N_{0,i} \ge 0$, $N_{0,i} \in {\mathbb {N}}$, for each federative unit *i*. The model identification is evaluated through an integral time-squared error performance criteria for reducing the contribution of the initial error of imported infections.

The nonlinear optimization problem in Equation (16) was solved for each federative unit *i* with IPOPT^52^ via CasADI/MATLAB^53^.

16 $$\begin{aligned} \begin{aligned} \min _{\mathbf {ident_i}}&\quad \sum _{k=N_{0,i}}^{N_f} (k-N_{0,i}+1) \left\Vert \mathbf {y_i}(k)-\mathbf {z_i}(k) \right\Vert _{\mathbf {Q_{id,i}}}^2\\ \text {Subject }&\text {to Equation}~(15)\text { and:}\\&\quad \mathbf {x_i}(k+1) = \mathbf {x_i}(k) + \int _{k}^{k+1} \mathbf {f}(\mathbf {x_i}(t),u_i(t)) \, d{t}\\&\quad \mathbf {y_i}(k) = \mathbf {h}(\mathbf {x_i}(k))\\&\quad S_i(t_{0,i}) \in [0.9,1],\ E_i(t_{0,i}) \in [0,0.1],\ \alpha _{0,i} \ge 0,\ a_{x_{a,i}} \in [0,0.1],\ a_{x_{s,i}} \in [0,0.1],\ x_{c,i} \in [0,1],\ x_{m,i} \in [0,1]\\&\quad a_{\zeta ,i}\ \in [0,1],\ b_{\zeta ,i} \in [0,0.25],\ c_{\zeta ,i} \ge 0,\ x_{a,i} \in [0,1],\ x_{s,i} \in [0,1],\ x_{s,i} + x_{c,i} \in [0,1],\ x_{a,i} \ge x_{s,i} \end{aligned} \end{aligned}$$

where $\mathbf {ident_i} = \left[ S_i(t_{0,i})\ E_i(t_{0,i})\ \alpha _{0,i}\ a_{x_{a,i}}\ a_{x_{s,i}}\ x_{c,i}\ x_{m,i}\ a_{\zeta ,i}\ b_{\zeta ,i}\ c_{\zeta ,i}\ w_{u,i}\right] ^T$, $\mathbf {z_i}$ are the measured variables and $\mathbf {Q_{id,i}} \in {\mathbb {R}}^{4\times 4}$ is a weight matrix calculated to normalize measurements from the early stages of the pandemic to July 2021 according to Equation (17). All numerical integration in the state estimators were solved with CVODES^54^ via CasADI/MATLAB. The initial guess was set as $\mathbf {ident_{0,i}} = [0.95\ 0.05\ 0.1\ 0.9\ 0.9\ 0.02\ 0.1\ 0.1\ 0.1\ 20\ 0.5]^T$. Results and location-dependent parameters are shown in Table 1.

17 $$\begin{aligned} \mathbf {Q_{{id,i}_{j,j}}} = \dfrac{1}{\left( y_{j,max}-y_{j,min}\right) ^2}, j \in \{1,2,3,4\} \end{aligned}$$

**Table 1:** Initial states and parameters of the proposed model for each federative unit *i*.

	AM	MS	RN	RS	RJ	SP
$t_{0,i}$	03 April 2020	19 April 2020	19 April 2020	19 April 2020	15 April 2020	27 March 2020
$S_i(t_{0,i})$	0.9449	0.9997	0.9951	0.9992	0.9762	0.9956
$E_i(t_{0,i})$	0.0551	0.0003	0.0049	0.0008	0.0238	0.0044
$\alpha _{0,i}$	0.1890	0.3953	0.5217	0.2848	0.2628	0.3223
$a_{x_{a,i}}$	1.0000	0.8999	1.0000	0.9999	1.0000	1.0000
$a_{x_{s,i}}$	0.9730	0.8999	0.9370	0.8963	0.9491	0.8861
$x_{c,i}$	0.0270	0.1001	0.0630	0.1037	0.0509	0.1139
$x_{m,i}$	0.3796	0.2586	0.4606	0.2833	0.4530	0.2694
$a_{\zeta ,i}$	0.2502	0.6539	0.6649	0.6419	0.2207	0.5591
$b_{\zeta ,i}$	0.2500	0.0900	0.1654	0.0429	0.2499	0.0540
$c_{\zeta ,i}$	39.3836	68.3508	51.2077	71.2191	38.0807	71.0390
${\tilde{\sigma }}_i$	0.0782	0.0859	0.0721	0.0822	0.0491	0.0794
${\tilde{\tau }}_i$	0.0672	0.0645	0.0766	0.0614	0.0726	0.0673
$w_u$	0.2604	1.000	1.0000	0.0000	0.7048	1.0000

## State and parameter estimation

State estimation is essential for a model with uncertainties and without measurements from all states. It comprehends estimates of unknown properties based on available measures while filtering them to reduce the noise effects. The proposed model in Equation (14) has $S_i$, $E_i$, $I_i$, and $A_i$ as unmeasurable, $Q_i$, $R_i$, and $H_{d,i}$ as unmeasured, and $T_i$, $D_i$, and $H_{t,i}$ as measured states. Besides, the sum of the states $Q_i,\ R_i,\ T_i,\ H_{d,i},$ $D_i$, and $H_{t,i}$, which represent the confirmed cases, is also a measured variable. Furthermore, the epidemiological model parameters have uncertainties related to time-varying NPI, its acceptance from the population, circulating virus variants, and vaccine coverage. Hence, a state estimator must accurately forecast the epidemiological evolution of COVID-19 on each analyzed federative unit *i*. In this work, we selected the parameters $\alpha _{0,i}$, $x_{c,i}$, and $x_{m,i}$ to estimate over time. Parameter estimation was performed using an augmented state $\mathbf {X_i}$ within a state estimator. The state $\mathbf {X_i}$ is defined as:

$$\begin{aligned} \mathbf {X_i} = \begin{bmatrix} \mathbf {x_i}\\ \psi _i \end{bmatrix} \end{aligned}$$

where $\psi _i = \left[ \alpha _{0,i}\ x_{c,i}\ x_{m,i} \right] ^T$ are the parameters to be estimated.

Time-varying dynamics from $\psi _i$ are unknown; thus, we assumed their differential equations equal to zero, and they are subject to artificial noise. Therefore, the state transition model $\mathbf {F}(\mathbf {x_i},u_i)$ for the augmented state is:

$$\begin{aligned} \mathbf {F}(\mathbf {X_i},u_i) = \begin{bmatrix} \mathbf {f}(\mathbf {x_i},u_i)\\ {\mathbf {0}} \end{bmatrix} \end{aligned}$$

Measurements in process control usually constraint real-time applicability for state estimators within seconds or minutes. Hence, the sampling time $T_s = 1$d allows analysis over different state estimation strategies. In this work, we evaluated the same scenario for each analyzed federative unit with a constrained extended Kalman filter (CEKF)^55^, a constrained extended Kalman filter and smoother (CEKF & S)^56^, and a moving horizon estimator (MHE)^57^. We used constrained observers to satisfy the feasible region ${\mathscr {X}}=\{{\mathbf {0}} \le \mathbf {x_i} \le {\mathbf {1}},\ H_{u,i} \in [0,1],\ \alpha _{0,i} \ge 0,\ x_{c,i} \in [0,1],\ x_{m,i} \in [0,1],\ x_{s,i} + x_{c,i} \in [0,1]\}$.

CEKF is an extension of the Kalman filter for nonlinear models. It uses a first-order Taylor expansion of the system model to estimate the current value based on the latest measurement and estimated state. The COVID-19 data, however, are given in weekly cycles in which weekends have fewer notifications that are updated on working days. The CEKF & S is an intermediate option between a regular CEKF and a MHE regarding computational time and performance. First, it forwards estimates from a moving horizon with a CEKF followed by backward estimation with a smoothing equation. The weekly oscillations are attenuated in the resulting state and in the covariance update. The MHE uses a moving horizon of estimates and measured variables in a nonlinear optimization problem, which is solved at each sampling time. This optimization problem has $N_p$ times the degrees of freedom of the CEKF, where $N_p$ is the horizon size. Therefore, it provides better estimation at the cost of a significantly higher computational time.

The error covariance matrix $\mathbf {P_{0,i}}$ and the initial estimated state $\mathbf {X_{0,i}}$ of each federative unit *i* are defined at $t_f=\text {1 October 2020}$. $\mathbf {x_{0,i}}$ and $\mathbf {y_{0,i}}$ can be found in Supplementary Table S3, while $\psi _{0,i}$ is shown in Table 1. The matrix $\mathbf {P_{0,i}}$ was defined as $\mathbf {P_{0,i}} = 10\ \text {diag} \left( \text {diag} \left( \left[ \mathbf {3 x_{0,i}^T}\ \psi _{0,i}^T \right] \left[ \mathbf {3 x_{0,i}^T}\ \psi _{0,i}^T \right] ^T \right) \right)$, the covariance matrix of process noise $\mathbf {Q_{k,i}}$ was defined as $\mathbf {Q_{k,i}}=\mathbf {P_{0,i}}$, and the covariance matrix of observation noise $\mathbf {R_{k,i}}$ was defined as $\mathbf {R_{k,i}} = 1000\ \text {diag} \left( \text {diag} \left( \mathbf {y_{0,i}}\ \mathbf {y_{0,i}^T} \right) \right)$.

Let us define the model with uncertainties:

18 $$\begin{aligned} \begin{aligned} \mathbf {X_i}(k|k-1)&= \mathbf {X_i}(k-1|k-1) + \int _{k-1}^{k} \mathbf {F}(\mathbf {X_i}(t),u_i(t)) \, d{t} + \omega _{k-1,i}\\ \mathbf {y_i} (k|k-1)&= \mathbf {h}(\mathbf {X_i}(k|k-1)) + \mathbf {v_{k,i}} \end{aligned} \end{aligned}$$

where $\omega _{k,i} \sim {\mathscr {N}}(\mathbf {0,Q_{k,i}})$ and $\mathbf {v_{k,i}}\sim {\mathscr {N}}(\mathbf {0,R_{k,i}})$ are the process and measurement noises, respectively.

The linearization of Equation (18) into a state-space model yields:

19a $$\begin{aligned} \mathbf {X_i}(k|k-1)&= \phi _{k-1,i}\ \mathbf {X_i} (k-1|k-1) \end{aligned}$$

19b $$\begin{aligned} \mathbf {y_i} (k|k-1)&= \mathbf {H_{k,i}\ X_i} (k|k-1) \end{aligned}$$

where the output matrix $\mathbf {H_{k,i}}$ and the state transition matrix $\phi _{k,i}$ are defined as:

19c $$\begin{aligned} \mathbf {H_{k,i}}&= \left( \dfrac{\partial \mathbf {h}(\mathbf {X_i}(k|k-1))}{\partial \mathbf {X_i}}\right) _{\mathbf {X_i}(k|k-1)} \end{aligned}$$

19d $$\begin{aligned} \phi _{k,i}&= \exp \left( \mathbf {G_{k,i}}\ T_s \right) \end{aligned}$$

19e $$\begin{aligned} \mathbf {G_{k,i}}&= \left( \dfrac{\partial \mathbf {F}(\mathbf {X_i}(k|k),u_i(k))}{\partial \mathbf {X_i}}\right) _{\mathbf {X_i}(k|k),u_i(k)} \end{aligned}$$

whose analytical expressions for these Jacobian matrices can be found in Supplementary Equation S1. We remark that Equation (19b) is equivalent to $\mathbf {h}(\mathbf {X_i}(k|k-1))$ as it is a linear function.

The initial conditions for each federative unit *i* were defined as $\mathbf {X_{0,i}} = \mathbf {X_i} (N_f|N_f)$, and $\mathbf {P_{0,i} = P_{N_f|N_f,i}}$ for all state estimators. All simulations with state estimation started at $t_{f} = \text {1 October 2020}$ and ended at $t_{sim} = t(N_{sim}) = \text {1 July 2021}$. The performance of the state estimation was evaluated using the mean absolute percentage error (MAPE) calculated for each output $\{y_j|j\in \{1,2,3,4\}\}$ as:

$$\begin{aligned} MAPE_j = \dfrac{100}{N_{sim}-N_{f}} \sum _{k=1}^{N_{sim}-N_{f}} \left| \dfrac{z_j(k)-y_j(k)}{z_j(k)} \right| \end{aligned}$$

### CEKF

For the sake of notation simplicity, the subscript *i*, denoting each federative unit, was suppressed from the description of the estimators. For the CEKF, the optimization problem in Equation (20) to update ${\mathbf {X}} (k|k)$ at each discrete time *k* corresponds to a quadratic programming, which was solved at each iteration with qpOASES^58^ via CasADI/MATLAB.

20 $$\begin{aligned} \begin{aligned} \min _{{\mathbf {X}} (k|k)}&\quad \left\Vert {\mathbf {y}} (k|k)-{\mathbf {z}} (k) \right\Vert _{\mathbf {R_k^{-1}}}^2 + \left\Vert {\mathbf {X}} (k|k)-{\mathbf {X}} (k|k-1) \right\Vert _{\mathbf {P_{k-1|k-1}^{-1}}}^2\\ \text {Subject to:}&\\&\quad {\mathbf {X}} (k|k-1) = {\mathbf {X}} (k-1|k-1) + \int _{k-1}^{k} {\mathbf {F}}({\mathbf {X}}(t),u(t)) d t\\&\quad {\mathbf {y}}(k|k) = \mathbf {H_k}\ {\mathbf {X}} (k|k)\\&\quad {\mathbf {X}} (k|k) \in {\mathscr {X}} \end{aligned} \end{aligned}$$

The state covariance matrix $\mathbf {P_{k|k}}$ was updated via the Riccati equation in discrete time as follows:

21 $$\begin{aligned} \begin{aligned} \mathbf {P_{k|k}} = \phi _{k-1} \mathbf {P_{k-1|k-1}} \phi _{k-1}^T - \phi _{k-1} \mathbf {P_{k-1|k-1}} \mathbf {H_k^T} \left[ \mathbf {H_k} \mathbf {P_{k-1|k-1}} \mathbf {H_k^T} + \mathbf {R_k} \right] ^{-1} \mathbf {H_k} \mathbf {P_{k-1|k-1}} \phi _{k-1}^T +\mathbf { Q_{k-1}} \end{aligned} \end{aligned}$$

Thereafter, the discrete-time is advanced to $k+1$, and Equations (20) and (21) are solved again to update ${\mathbf {X}} (k|k)$ and $\mathbf {P_{k|k}}$.

### CEKF & S

The CEKF & S was implemented according to the formulation from Salau et al.^56^ The state estimation was initially done $N_{p}-1$ times with the CEKF from the previous section.

The Rauch-Tung-Stribel (RTS) smooth equations^59^ were applied from $t(N_p)$ to the simulation end $(t_{sim})$. Each discrete time started with an additional CEKF iteration to calculate ${\mathbf {X}} (k|k)$ and $\mathbf {P_{k|k}}$. Let us define $\mathbf {X^S} (k) = {\mathbf {X}} (k|k)$, $\mathbf {P_k^S}=\mathbf {P_{k|k}}$, ${\tilde{\mathbf {P}}}_{\mathbf {k|k}}= \left[ \mathbf {P_{k-N_{p}|k-N_{p}}^T}\ \mathbf {P_{k-N_{p}+1|k-N_{p}+1}^T}\ \cdots \ \mathbf {P_{k|k}^T} \right] ^T$, and $\tilde{{\mathbf {X}}} (k|k) = \left[ {\mathbf {X}} (k-N_{p}|k-N_{p})^T\ {\mathbf {X}} (k-N_{p}+1|k-N_{p}+1)^T\ \cdots \ {\mathbf {X}} (k|k)^T \right] ^T$ for estimating backward with the Rauch-Tung-Striebel (RTS) smooth equations^59^. Solving Equation (22) for $\{j \in [1, N_{p}]|j\in {\mathbb {N}}\}$, yields the solution $\mathbf {X^S}(k-N_p)$ and $\mathbf {P^S_{k-N_p}}$, which is the initial conditions ${\mathbf {X}} (k-N_{p}|k-N_{p}) = \mathbf {X^S} (k-N_{p})$ and $\mathbf {P_{k-N_{p}|k-N_{p}}} =\mathbf {P^S_{k-N_{p}}}$ for forward estimation until the current step *k* through $N_p$ iterations of the CEKF.

22 $$\begin{aligned} \begin{aligned} {\mathbf {X}} (k+1-j|k-j)&= {\mathbf {X}} (k-j|k-j) + \int _{k-j}^{k+1-j} \mathbf {F}(\mathbf {X}(t),u(t)) d t\\ \mathbf {P_{k+1-j|k-j}}&= \phi _{k-j} \mathbf {P_{k-j|k-j}} \phi _{k-j}^T + \mathbf {Q_{k-j}}\\ \mathbf {C_{k-j}}&= \mathbf {P_{k-j|k-j}} \phi _{k-j}^T \left[ \mathbf {P_{k+1-j|k-j}} \right] ^{-1}\\ \mathbf {X^S} (k-j)&= {\mathbf {X}} (k-j|k-j) + \mathbf {C_{k-j}} \left[ \mathbf {X^S} (k+1-j) - {\mathbf {X}} (k+1-j|k-j) \right] \\ \mathbf {P^S_{k-j}}&= \mathbf {P_{k-j|k-j}} + \mathbf {C_{k-j}}\left[ \mathbf {P^S_{k+1-j}} - \mathbf {P_{k+1-j|k-j}} \right] \mathbf {C_{k-j}^T}\\ \end{aligned} \end{aligned}$$

State and covariance estimations from each step are used to update their respective values in the vectors $\tilde{{\mathbf {X}}}(k|k)$ and ${\tilde{\mathbf {P}}}_{\mathbf {k|k}}$ (k|k). Two horizon sizes $N_{p} = 7$ and $N_{p} = 28$ were used in the simulations to evaluate the effect of $N_p$ on the estimator performance.

### MHE

The MHE was implemented according to the formulation from Rawlings et al.^60^ The past horizon $N_{p}=\min \left( k-N_f,N_{p,0}\right)$ at each discrete-time *k*, where $N_{p,0}$ is the given horizon size for the estimator. Hence, we can set the initial condition for the optimization problem in Equation (23) as $\mathbf {P_{k-N_{p}-1|k-N_{p}-1}}$ and ${\mathbf {X}} (k-N_{p}-1|k-1)$. The state is updated at each discrete time *k* with the solution of Equation (23) through IPOPT^52^ via CasADI/MATLAB.

23 $$\begin{aligned} \begin{aligned} \min _{{\mathbf {X_k}}}&\quad \left\| \hat{{\mathbf {X}}} (k-N_{p}|k) -{\mathbf {X}} (k-N_{p}|k) \right\| _{\mathbf {P_{k-N_{p}-1|k-N_{p}-1}^{-1}}}^2 + \sum _{j=k-N_{p}+1}^{k} \left\| \hat{{\mathbf {X}}} (j|k) -{\mathbf {X}} (j|k) \right\| _{\mathbf {Q_{j-1}^{-1}}}^2 \\&\quad + \sum _{j=k-N_{p}}^{k} \left\| {\mathbf {y}} (j|k)-{\mathbf {z}} (j) \right\| _{\mathbf {R_j^{-1}}}^2\\ \text {Subject to:}&\\&\quad {\mathbf {X}} (j|k) = \hat{{\mathbf {X}}} (j-1|k) + \int _{j-1}^{j} {\mathbf {F}}({\mathbf {X}}(t),u(t)) d t\\&\quad {\mathbf {y}}(j|k) = \mathbf {H_j}\ {\mathbf {X}} (j|k)\\&\quad {\hat{{\mathbf {X}}}}(j|k) \in {\mathscr {X}}\\&\quad j \in [k-N_{p},k],\ j\in {\mathbb {N}} \end{aligned} \end{aligned}$$

where $\mathbf {X_k} = \left[ \hat{{\mathbf {X}}} (k-N_{p}|k)^T\ \hat{{\mathbf {X}}} (k-N_{p}+1|k)^T\ \cdots \ \hat{{\mathbf {X}}} (k|k)^T \right] ^T$ are the estimated states and $\hat{{\mathbf {X}}} (k-N_{p}-1|k) = {\mathbf {X}} (k-N_{p}-1|k-1)$. The state covariance matrix $\mathbf {P_{k|k}}$ is updated via the Riccati Eqs. (21).

The MHE gives estimations over a horizon $N_p$ based on the initial conditions $\mathbf {P_{k-N_{p}-1|k-N_{p}-1}}$ and ${\mathbf {X}} (k-N_{p}-1|k-1)$. Current estimations at a discrete time *k* are $\hat{{\mathbf {X}}} (k|k)$ and $\mathbf {P_{k|k}}$. The advance in discrete time is carried out by solving Equations (23) and (21) based on previous estimations from $k-N_{p}-1$ to $k-1$. The MHE was implemented with $N_{p,0} = 7$.

## Simulation results and discussion

**Figure 2:**
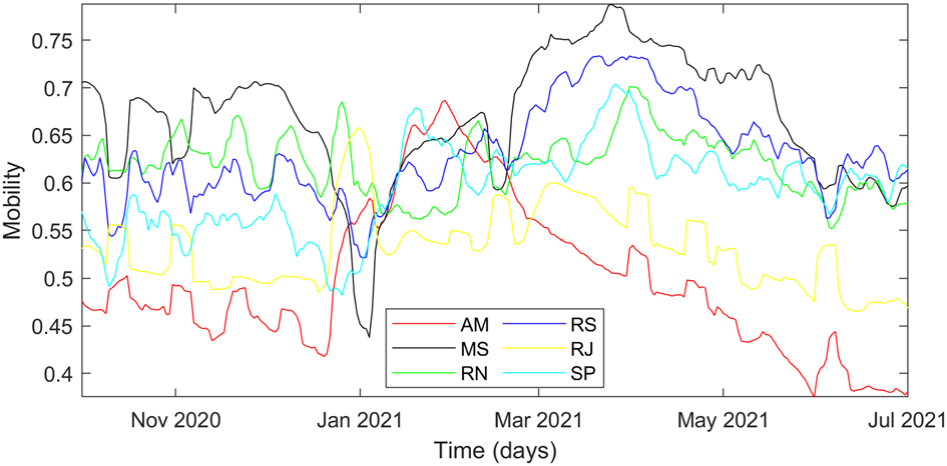
Time evolution of input variable related to social distancing.

In this section, we present the results from simulations for federative units Amazonas (AM), Mato Grosso do Sul (MS), Rio Grande do Norte (RN), Rio Grande do Sul (RS), Rio de Janeiro (RJ) and São Paulo (SP). States and parameters were estimated from 1 October 2020 to 1 July 2021. Confirmed cases ($y_1$) and deceased ($y_2$) measures were obtained from the Ministry of Health of Brazil^2^, whereas hospitalized ($y_3$) and healed with treatment ($y_4$) were obtained from the Brazilian SARS database^38,39^. The input variable $u_i$, calculated using Google mobility data, is presented in Fig. 2 for each federative unit.

**Figure 3:**
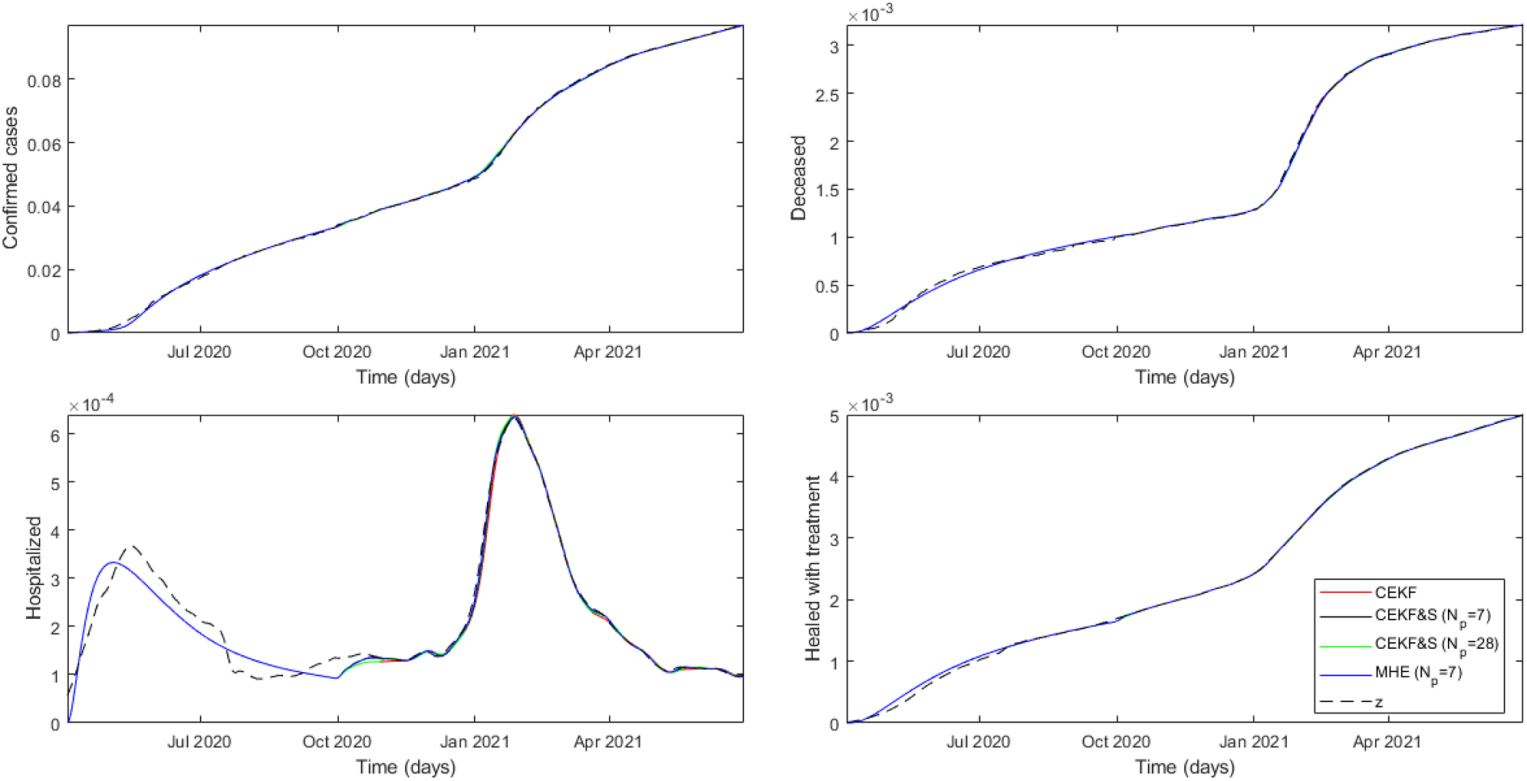
Time evolution of measures and estimated outputs from Amazonas (AM).

All three state estimators drove the estimation toward the measure $\mathbf {z_i}$ for each federative unit, as shown in Table 2 with MAPE results smaller than 5%. Hence, COVID-19 dynamic evolution on regional populations was captured despite the model assumptions. The tuning of $\mathbf {P_{0,i}}$ and $\mathbf {Q_{k,i}}$ based on $\psi _{0,i}$ values resulted in better estimates of $y_2$ and $y_4$, and higher estimation error on $y_3$ for all studied cases. Using the same tuning formulation for all analyzed federative units implied some suboptimal sets of tuning parameters. Table 2 lets us identify the worst estimation from CEKF followed by CEKF & S and MHE according to expectations. Moreover, the increase in horizon size $N_p$ from 7 to 28 showed loss of estimation accuracy of the CEKF & S. The lack of long-term correlation for estimating state and parameter backward is probably a cause for this result; however, additional studies are required to verify the existence of other sources. The time evolution of output measures from all federative units can be found in Supplementary Figs. S1–S5, except for Amazonas, which is shown in Fig. 3.

**Table 2:** Mean absolute percentage error for simulation with state estimation for each federative unit *i*.

	State Estimator	AM	MS	RN	RS	RJ	SP
$y_1$	CEKF	0.40	0.59	1.05	1.49	0.56	0.61
	CEKF & S ($\hbox {N}_p = 7$)	0.34	0.51	0.90	1.10	0.51	0.52
	CEKF & S ($\hbox {N}_p = 28$)	0.45	0.56	0.98	1.19	0.66	0.64
	MHE ($\hbox {N}_p = 7$)	0.32	0.45	0.85	0.99	0.49	0.48
$y_2$	CEKF	0.37	0.42	0.31	0.35	0.36	0.29
	CEKF & S ($\hbox {N}_p = 7$)	0.34	0.37	0.28	0.32	0.35	0.28
	CEKF & S ($\hbox {N}_p = 28$)	0.38	0.39	0.30	0.33	0.36	0.28
	MHE ($\hbox {N}_p = 7$)	0.34	0.33	0.27	0.30	0.32	0.25
$y_3$	CEKF	3.94	3.57	2.05	3.66	1.98	2.35
	CEKF & S ($\hbox {N}_p = 7$)	3.14	2.48	1.55	2.53	1.52	1.60
	CEKF & S ($\hbox {N}_p = 28$)	3.58	2.55	1.45	2.57	1.62	1.55
	MHE ($\hbox {N}_p = 7$)	2.52	1.66	1.26	2.07	1.06	1.25
$y_4$	CEKF	0.21	0.21	0.21	0.17	0.11	0.12
	CEKF & S ($\hbox {N}_p = 7$)	0.20	0.18	0.16	0.15	0.11	0.11
	CEKF & S ($\hbox {N}_p = 28$)	0.25	0.19	0.16	0.17	0.14	0.13
	MHE ($\hbox {N}_p = 7$)	0.18	0.16	0.15	0.16	0.10	0.11

**Figure 4:**
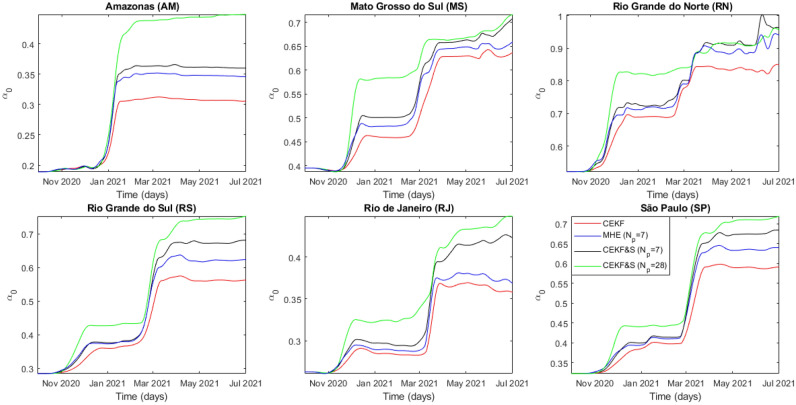
Time evolution of estimated contagion rate $\alpha _0$ from all analyzed federative units.

**Figure 5:**
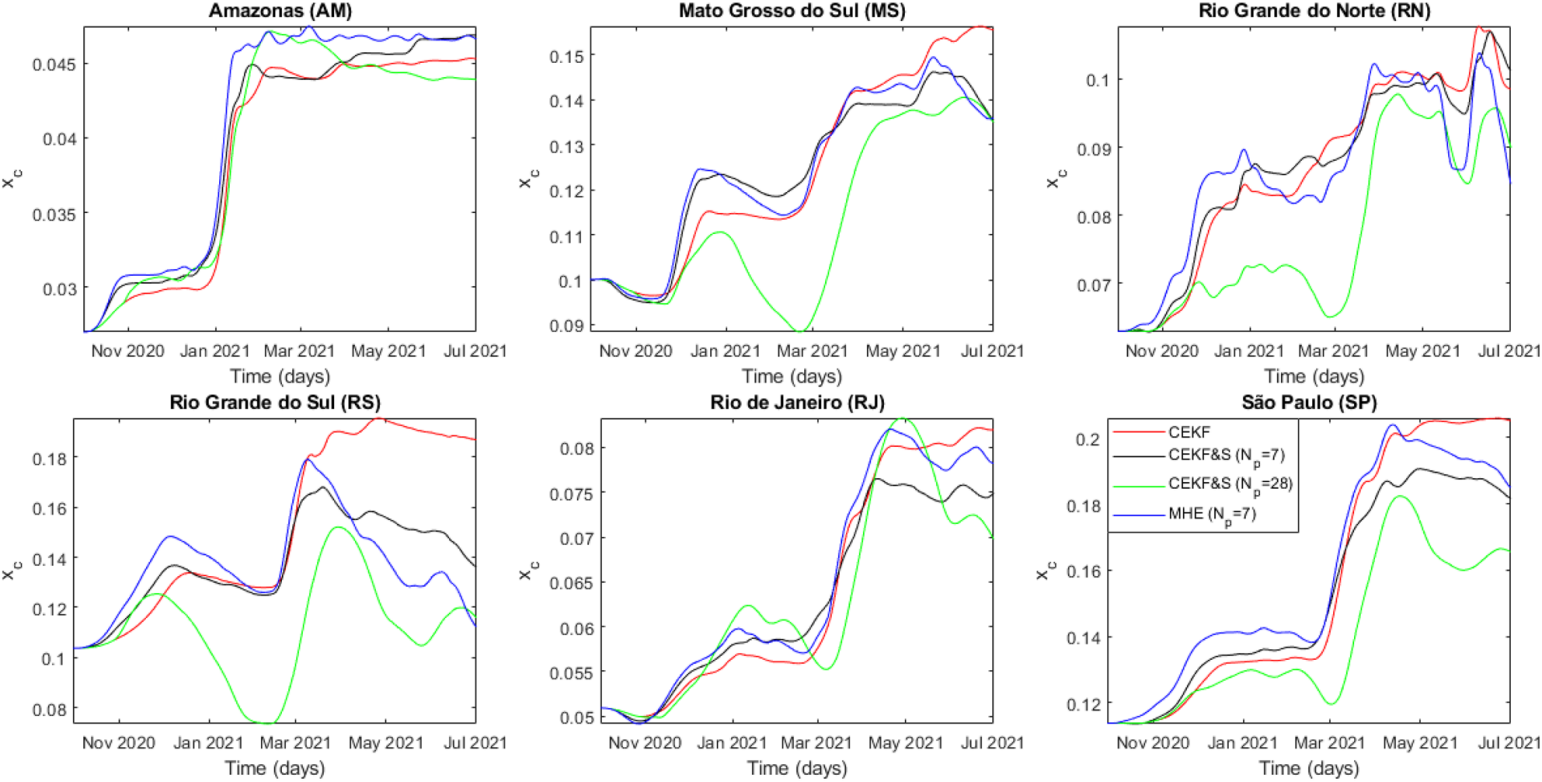
Time evolution of estimated fraction of symptomatic individuals who develop mild and severe symptoms $x_c$ from all analyzed federative units.

**Figure 6:**
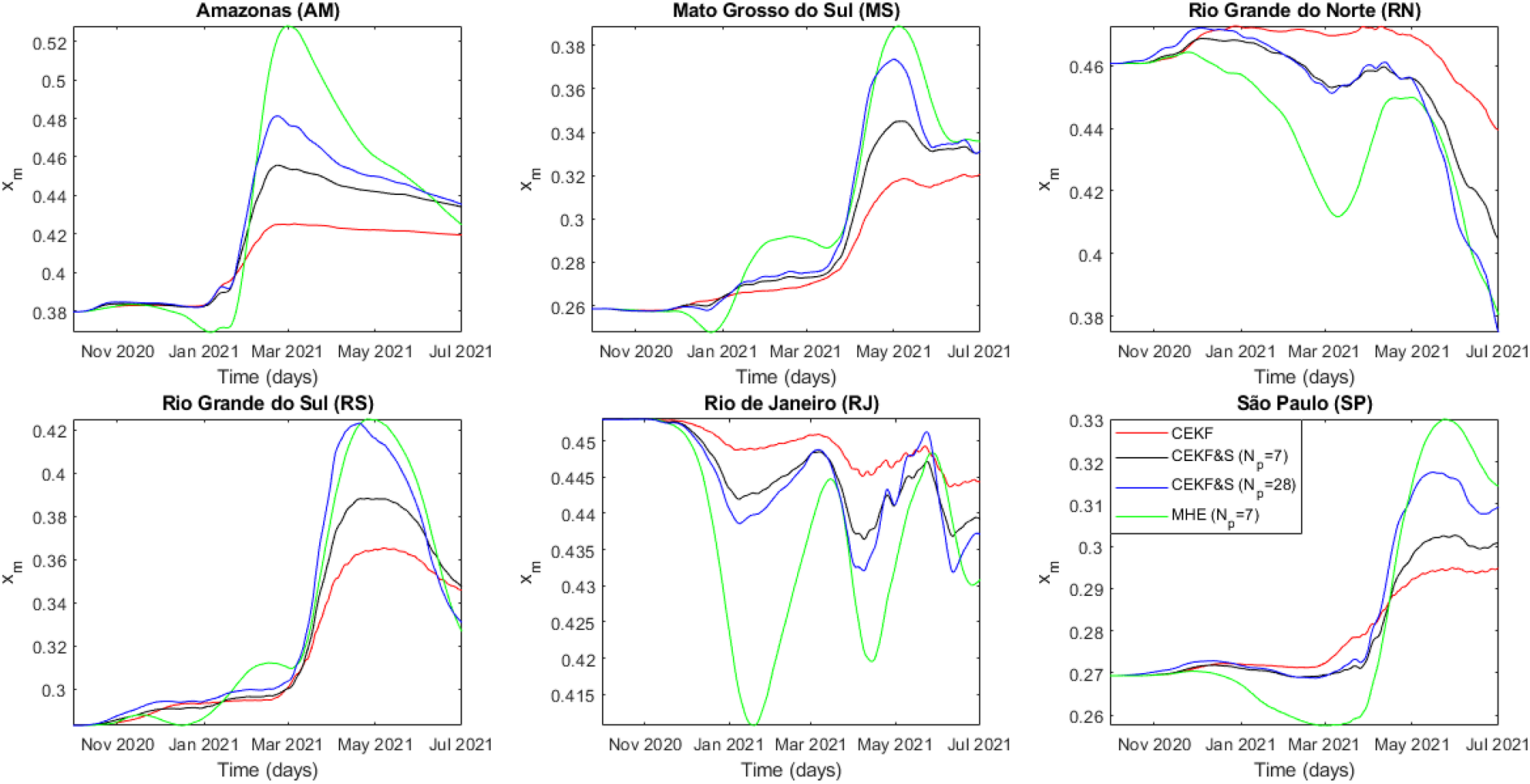
Time evolution of estimated fraction of threatened individuals who decease $x_m$ from all analyzed federative units.

Amazonas had only two coronavirus waves identifiable through $y_3$, as can be seen in Fig. 3, unlike other analyzed federative units. GISAID data^7^ indicated that variants B.1.1.33, B.1.1.28, and a local B.1.378 were significantly circulating from 1 October 2020 to 4 December 2020 when the first Gamma variant sequence was identified. Hence, the predominant circulation of the Zeta variant starting in mid-October 2020, was quickly overlapped by the Gamma variant resulting in a single wave. The estimated parameters, presented in Figs. 4-6, represent this profile specifically with MHE estimations and indicate that CEKF & S with $N_p=7$ had closer parameter estimations with MHE than CEKF & S with $N_p=28$. It is important to emphasize that MHE was applied with $N_p = 7$, which could explain this similarity.

All other federative units also had an overlap of the Zeta and Gamma waves; however, there were higher periods with the circulation of the Zeta variant. Figs. 4–6 infer rougher parameter estimation from CEKF & S with $N_p=28$, despite having a larger horizon. The moving horizon with estimated states backward and forward might smooth estimations overall, but each estimate still considers a single measure. Further studies on the effects of $N_p$ in CEKF & S are required, but these were not the subject of this work. Since MHE provided better state and parameter estimations, descriptions henceforth are related to these estimates.

Age distribution and local healthcare do not explain the discrepancy between $x_{c,i}$ and $x_{m,i}$ among the analyzed federative units observed in Figs. 5 and 6. This discrepancy points out the violation of the model assumption considering the complete identification of hospitalized individuals infected by COVID-19. Some case studies presented higher $x_{c,i}$ altogether with lower $x_{m,i}$, which do not affect the infection fatality rate (IFR) but indicate underreporting of COVID-19 cases among hospitalized individuals. Amazonas, Rio Grande do Norte, and Rio de Janeiro presented testing policies focused on more severe hospitalizations. The IFR calculus defined in Equation (24) highlights this dynamic.

24 $$\begin{aligned} IFR_i = (1-p) x_{c,i} x_{m,i} \end{aligned}$$

**Figure 7:**
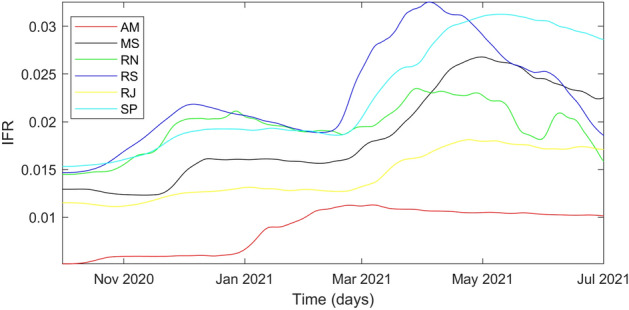
Time evolution of IFR considering estimated parameters.

The results in Fig. 7 indicate a guaranteed underreporting of deaths in Amazonas. In addition to Figs. 5 and 6, the $x_{m,i}$ decrease shows that underreporting of hospitalized individuals in Rio de Janeiro and Rio Grande do Norte reduced over time. The lethality evaluation of a variant by $x_{m,i}$ is unfeasible because it decreased in Rio Grande do Norte and Rio de Janeiro due to testing policy. In addition, its peaks in Mato Grosso do Sul, Rio Grande do Sul, and São Paulo are explained by delayed notifications after an overload of the health system, which temporarily increases mortality. Hence, we used $IFR_i$ to conclude that the Zeta variant increased lethality from $11\%$ in Rio de Janeiro to $30\%$ in Rio Grande do Norte based on $IFR_i(t_{0,i})$ calculated with values from Table 1. In addition, $\alpha _{0,i}$ estimation indicates that the Zeta transmissibility increased from $10\%$ in Rio de Janeiro to $37\%$ in Rio Grande do Norte.

Amazonas faced oxygen shortage on the Gamma variant wave, which implicated in mortality increase beyond virus mutations. Nonetheless, estimates of $\alpha _{0,i}$ had an increase of $84\%$ over its initial value, while $x_{c,i}$ had an increase of $67\%$. In addition, its estimations on $x_{c,i}$ are smoother, which allowed identifying the increase in the severity of the disease ranging from $36 \%$ in Rio Grande do Sul to $71\%$ in São Paulo. The analysis through $IFR_i$ indicated a lethality increase between $44\%$ in Rio Grande do Norte and $107\%$ in Amazonas for the Gamma variant. Moreover, transmissibility increased between $43\%$ in Rio de Janeiro and $119 \%$ in Rio Grande do Sul based on first-wave values of $\alpha _{0,i}$ from Table 1. Further investigation on $IFR_i$ points out variant spread countrywide, being Amazonas its source. The Gamma variant spread initially to the farthest case study from Amazonas: Rio Grande do Sul. Afterward, it spread to the Brazilian economic center, São Paulo, and thereafter to the rest of the federative units at a similar rate. The quicker propagation of the Gamma variant to the farthest location from Amazonas, Rio Grande do Sul, is explained by a less rigid NPI highlighted by the highest $\alpha _{0,i}$ estimate. São Paulo had only the fourth highest $\alpha _{0,i}$ among the studied cases. However, it was the second to significantly contract the Gamma variant, which enforces the theory that it spread countrywide afterward and corroborates its classification as a super-spreader city par excellence by Nicolelis et al.^35^ Overall, $\alpha _{0,i}$ more clearly indicated the emergence of circulating variants in the system. Finally, $IFR_i$ decreased in later times for most federative units until 1 July 2021, indicating that vaccination coverage does reduce mortality in infected individuals.

The circulating variant dynamics assumed the unique circulation of the lineage, whose uncertainty was reduced by a manual definition of the analysis period for each variant. Most studied cases had the Zeta wave overlapped by the Gamma variant; thus, dynamic estimations are expected to be lower or equal to the actual value. The Zeta evaluation period was defined in the last 15 days before Gamma variant emergence. The Gamma variant estimations are expected to be more accurate since it was predominant over some time for all cases studies. The Gamma evaluation period was defined from the first stationary point after variant emergence to the end of the simulation.

The computational time of the three state estimators was evaluated throughout the average simulation time among all analyzed federative units for 273 d, from 1 October 2020 to 1 July 2021. Simulation time was measured by the tic and toc functions in MATLAB. All simulations were carried out on an AMD Ryzen 5 5600X 3.70 GHz in a sequence to mitigate computational noises. The average simulation time was 25.4 s for CEKF, 136.7 s for CEKF & S with $N_p = 7$, 498 s for CEKF & S with $N_p = 28$, and 10265 s for MHE. Even the average execution time of 38 s per sampling time for the MHE implies real-time applicability of the state estimators with the selected tuning in the COVID-19 pandemic scenario since all of them have execution times lower than the sampling time $T_s = 1$ d. CEKF & S performance and computational time were between the CEKF and the MHE, which enforce it as an alternative for processes with faster sampling times.

The definition of a compartmental model inherits limitations regarding the closed system and homogeneous compartment assumptions. In addition, all numerical results are dependent on the initial condition, which was determined from a nonlinear optimization in this work. Age distribution was neglected in the model formulation to aim for real-time applicability and fulfill available data of confirmed cases. Model assumptions uncertainties are mitigated by the state and parameter estimation; however, they do not guarantee realistic estimations. For instance, the mitigation of the variants reinfection mostly through compartments $S_i$ and $H_{u,i}$ instead of estimated parameters $\alpha _{0,i}$, $x_{c,i}$, $x_{m,i}$ is a consequence of the tuning. Hence, a fine-tuning procedure may be required for severe assumption violations to avoid unrealistic estimations. Mitigation of multiple uncertainties in the model formulation is achieved by a conservative tuning concerning small dynamic changes. Hence, smooth vaccination coverage and gene sequence dynamics might be noise to the estimator. Data quality also limits a more aggressive tuning for state estimators, such as sudden data updates of underreporting for cases and deceased (e.g., Rio Grande do Norte on 23 July 2021). Nonetheless, the proposed method allowed the study of overall dynamics in each studied case.

## Conclusion

In this work, we proposed a mathematical model able to identify underreported cases of COVID-19 from hospitalized and deceased individuals by comparing the fraction of symptomatic individuals who develop severe or mild symptoms, the fraction of threatened individuals who decease, and the infection fatality rate among analyzed federative units. In addition, the model identified circulating variant dynamics in the aforementioned parameters, and characterize them under some assumptions. We remark that this model is suitable for control strategies, assuming there are available hospitalized data.

The performance among estimators confirmed MHE as a more suitable state estimator for COVID-19 due to daily sampling time. Nonetheless, CEKF & S presented reasonable estimations for comparison, and a significant reduction in computational time, which make it applicable in real-time applications.

Parameter estimations identified a lethality increase ranging from 11 to $30\%$ and a transmissibility increase between 10 and $37\%$ for the Zeta mutation. In addition, we found that the Gamma mutations caused a lethality increase ranging from 44 to $107\%$ and a transmissibility increase between 43 and $119\%$. The estimation strategy successfully detected and estimated dynamics affected by the emergence of COVID-19 variants, which improves model accuracy for further predictions. Moreover, an initial decrease in lethality due to vaccination was also observed. Hence, the parameter estimation within recursive state estimation can deal with dynamic uncertainties from the COVID-19 pandemic.

Future works account for implementing an economic model predictive control and studies on inserting vaccination into the proposed model. Delta variant has been predominant in Brazil since August 2021. It was disregarded from an initial analysis because its mutation highly increases contagion among vaccinated people, which are measured. Therefore, a model comprising vaccinated individuals should generate better estimations of Delta dynamics.

## Supplementary Information

Supplementary Information.

## Data Availability

The data sets used in this study are publicly available in the Ministry of Health of Brazil (https://covid.saude.gov.br/)^2^; Brazilian SARS database (https://opendatasus.saude.gov.br/dataset/srag-2020 and https://opendatasus.saude.gov.br/dataset/srag-2021-e-2022)^38,39^; and Google LLC (https://www.google.com/covid19/mobility/)^46^.
