# Supplementary material for: Recursive state and parameter estimation of COVID-19 circulating variants dynamics
Source: Sci Rep. 2022 Sep 23;12:15879. doi: 10.1038/s41598-022-18208-6 (PMC9508243; doi:10.1038/s41598-022-18208-6)
Supplement: Supplementary file 1 — Supplementary Information. [file 41598_2022_18208_MOESM1_ESM.pdf]

Supplementary Material

Recursive state and parameter estimation of COVID-19 circulating variants dynamics

Authors: Daniel M. Silva* and Argimiro R. Secchi

Affiliation: Chemical Engineering Program/COPPE, Universidade Federal do Rio de Janeiro, Rio de Janeiro, 21941-942, Brazil

Correspondence to: dmsilva@peq.coppe.ufrj.br

Supplementary Tables

Table S1. Cross-correlation matrix with zero lag from Google mobility data.

	Recreation	Essentials	Parks	Transit	Workplace	Resident
Recreation	1	-0.0223	0.9180	0.9450	0.7425	-0.9370
Essentials	-0.0223	1	-0.0998	0.0336	0.3950	0.2681
Parks	0.9180	-0.0998	1	0.8550	0.5862	-0.8788
Transit	0.9450	0.0336	0.8550	1	0.7641	-0.8931
Workplace	0.7425	0.3950	0.5862	0.7641	1	-0.6644
Resident	-0.9370	0.2681	-0.8788	-0.8931	-0.6644	1

Table S2. Prevalence bounds from seroprevalence survey EPICOVID19-BR.

	AM	MS	RN	RS	RJ	SP
$Prev_{i,1,min}$	0.0800	0	0	0	0	0.0010
$Prev_{i,1,max}$	0.1600	0.0220	0.0420	0.0087	0.0370	0.0430
. $Prev_{i,2,min}$	0.1000	0	0.0090	0	0.0250	0
$Prev_{i,2,max}$	0.1800	0.0120	0.0570	0.0093	0.0890	0.0280
$Prev_{i,3,min}$	0.0440	0	0.0240	0.0001	0.0420	0
$Prev_{i,3,max}$	0.1100	0.0140	0.0760	0.0093	0.1200	0.0190

Table S3. Initial states for the state estimation for each federative unit i.

	AM	MS	RN	RS	RJ	SP
$S_i(t_f)$	0.7873	0.955	0.953	0.966	0.899	0.946
$L_i(t_f)$	0.00233	0.00130	0.000244	0.000645	0.00197	0.000640
$I_i(t_f)$	0.00280	0.000723	0.000148	0.000444	0.00222	0.000501
$D_i(t_f)$	0.00103	0.00182	0.000390	0.00102	0.000618	0.000784
$A_i(t_f)$	0.00274	0.000743	0.000142	0.000412	0.00214	0.000462
$R_i(t_f)$	0.00100	0.00158	0.000368	0.000931	0.000598	0.000715
$T_i(t_f)$	0.000092	0.000195	0.0000287	0.000147	0.000128	0.000129
$H_{d,i}(t_f)$	0.02859	0.0189	0.0164	0.0121	0.0117	0.0157
$E_i(t_f)$	0.00100	0.000445	0.000652	0.000416	0.00103	0.000755
$H_{t,i}(t_f)$	0.00164	0.00128	0.000763	0.00105	0.00123	0.00205

Supplementary Equations

$$
\mathbf{G_{k,i}} = \begin{bmatrix}
-\nu_i & 0 & -\alpha_i S_i & 0 & -\alpha_i S_i & 0 & 0 & 0 & 0 & 0 & -c_2 & 0 & 0 \\
\nu_i & -\rho & \alpha_i S_i & 0 & \alpha_i S_i & 0 & 0 & 0 & 0 & 0 & c_2 & 0 & 0 \\
0 & p\rho & -(\lambda_i+\varepsilon_i) & 0 & 0 & 0 & 0 & 0 & 0 & 0 & 0 & 0 & 0 \\
0 & 0 & \varepsilon_i & -\lambda_i & 0 & 0 & 0 & 0 & 0 & 0 & 0 & 0 & 0 \\
0 & (1-p)\rho & 0 & 0 & -\theta_i - c_1 & 0 & 0 & 0 & 0 & 0 & 0 & -(c_3+c_4)A_i & 0 \\
0 & 0 & 0 & 0 & \theta_i & -c_1 & 0 & 0 & 0 & 0 & 0 & c_3 A_i - c_4 R_i & 0 \\
0 & 0 & 0 & 0 & \mu_i & \mu_i & -(\sigma_i+\tau_i) & 0 & 0 & 0 & 0 & c_5 & x'_{e,i}(\tilde{\sigma}_i - \tilde{\tau}_i)T_i \\
0 & 0 & 0 & \lambda_i & 0 & \kappa_i & \sigma_i & 0 & 0 & 0 & 0 & x'_{k,i}\tilde{\kappa} R_i & 0 \\
0 & 0 & 0 & 0 & 0 & 0 & \tau_i & 0 & 0 & 0 & 0 & 0 & x'_{e,i}\tilde{\tau}_i T_i \\
0 & 0 & 0 & 0 & 0 & 0 & \sigma_i & 0 & 0 & 0 & 0 & 0 & -x'_{e,i}\tilde{\sigma}_i T_i \\
0 & 0 & 0 & 0 & 0 & 0 & 0 & 0 & 0 & 0 & 0 & 0 & 0 \\
0 & 0 & 0 & 0 & 0 & 0 & 0 & 0 & 0 & 0 & 0 & 0 & 0 \\
0 & 0 & 0 & 0 & 0 & 0 & 0 & 0 & 0 & 0 & 0 & 0 & 0
\end{bmatrix} \tag{S1a}
$$

$$
\mathbf{H_{k,i}} = \begin{bmatrix}
0 & 0 & 0 & 1 & 0 & 1 & 1 & 1 & 1 & 1 & 0 & 0 & 0 \\
0 & 0 & 0 & 0 & 0 & 0 & 0 & 0 & 1 & 0 & 0 & 0 & 0 \\
0 & 0 & 0 & 0 & 0 & 0 & 1 & 0 & 0 & 0 & 0 & 0 & 0 \\
0 & 0 & 0 & 0 & 0 & 0 & 0 & 0 & 0 & 1 & 0 & 0 & 0
\end{bmatrix} \tag{S1b}
$$

where $c_1 = \mu_i + \kappa_i$, $c_2 = (1-u_i)(I_i + A_i)S_i$, $c_3 = x_{\theta,i}\tilde{\varepsilon}$, $c_4 = (-x'_{k,i} - x'_{\theta,i})\tilde{\mu} + x'_{k,i}\tilde{\kappa}$, $c_5 = (1 - x'_{k,i} - x'_{\theta,i})\tilde{\mu}(A_i + R_i)$, and:

$$
x'_{\theta,i} = \frac{-x_{s,i}\tilde{\varepsilon}\tilde{\kappa}\tilde{\mu}\left(\tilde{\kappa} + x_{s,i}(\tilde{\mu} - \tilde{\kappa})\right)}{\left((1 - x_{c,i} - x_{s,i})\tilde{\kappa}\tilde{\mu} + (1 - x_{c,i})x_{s,i}\tilde{\varepsilon}\tilde{\mu} + x_{c,i}x_{s,i}\tilde{\varepsilon}\tilde{\kappa}\right)^2} \tag{S2}
$$

$$
x'_{k,i} = \frac{x_{s,i}x_{\theta,i}(\tilde{\varepsilon} - \tilde{\mu})}{\tilde{\kappa} + x_{s,i}(\tilde{\mu} - \tilde{\kappa})} \tag{S3}
$$

$$
x'_{e,i} = \frac{\tilde{\sigma}_i\tilde{\tau}_i}{\left((1 - x_{m,i})\tilde{\tau}_i + x_{m,i}\tilde{\sigma}_i\right)^2} \tag{S4}
$$

Supplementary Figures

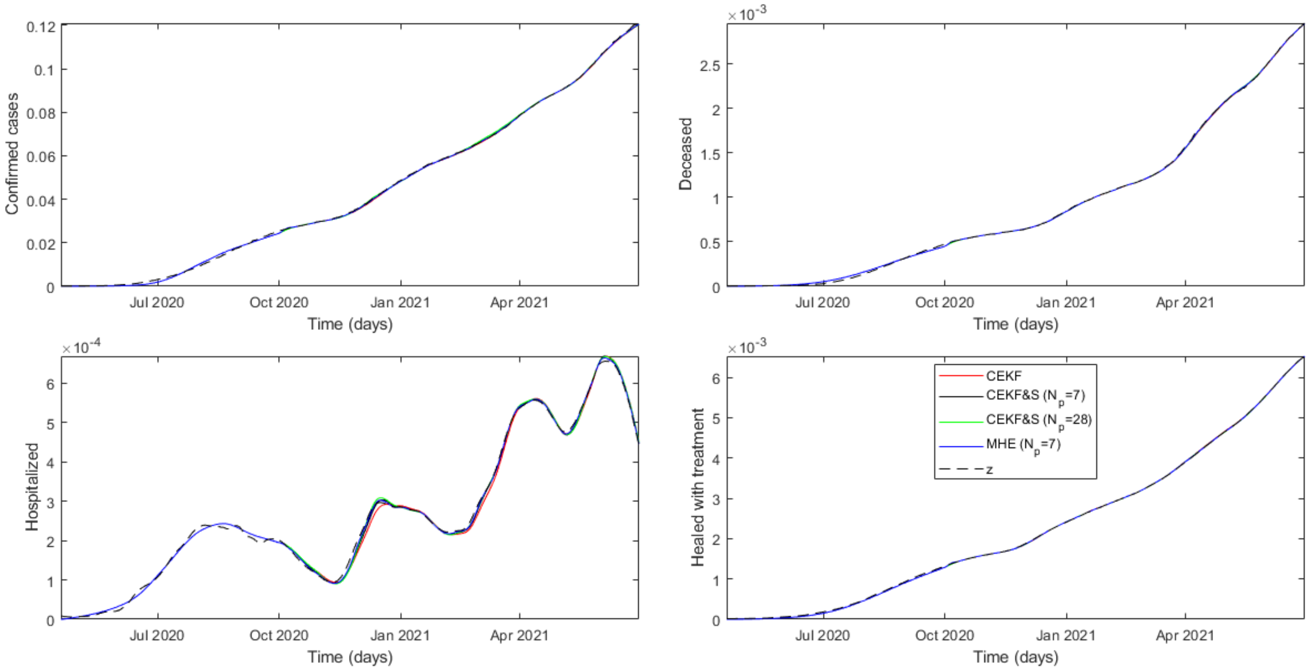

Figure S1. Time evolution of measures and estimated outputs from Mato Grosso do Sul (MS).

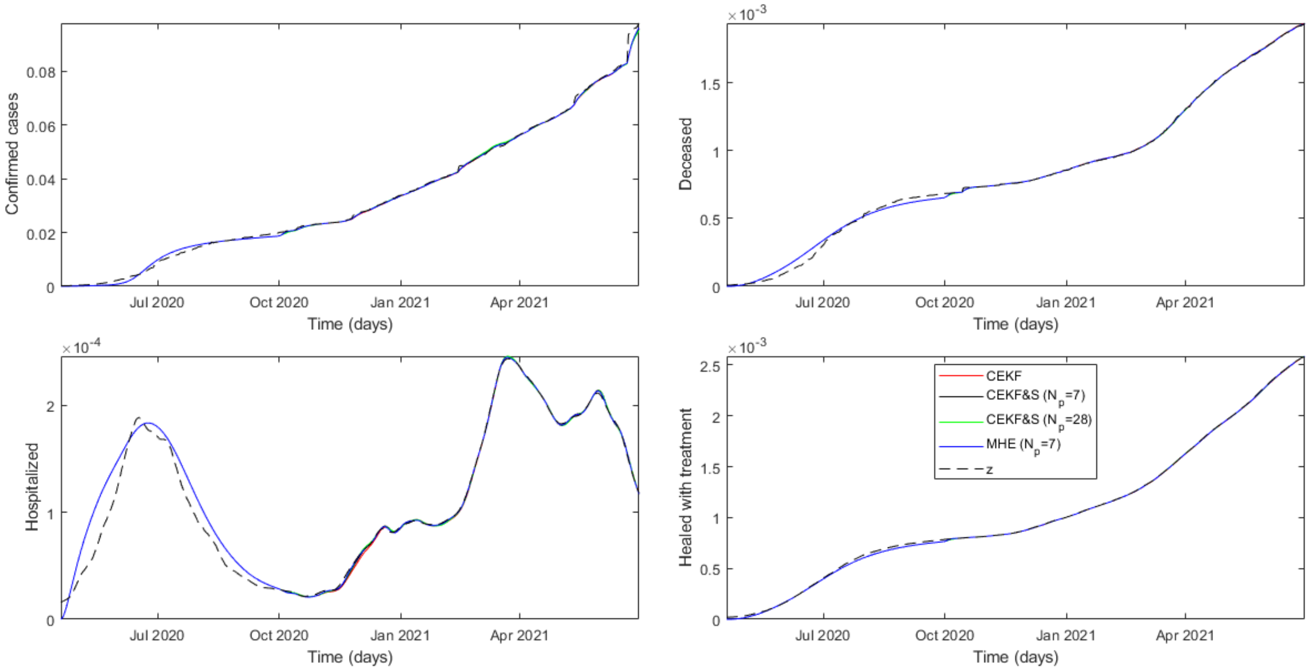

Figure S2. Time evolution of measures and estimated outputs from Rio Grande do Norte (RN).

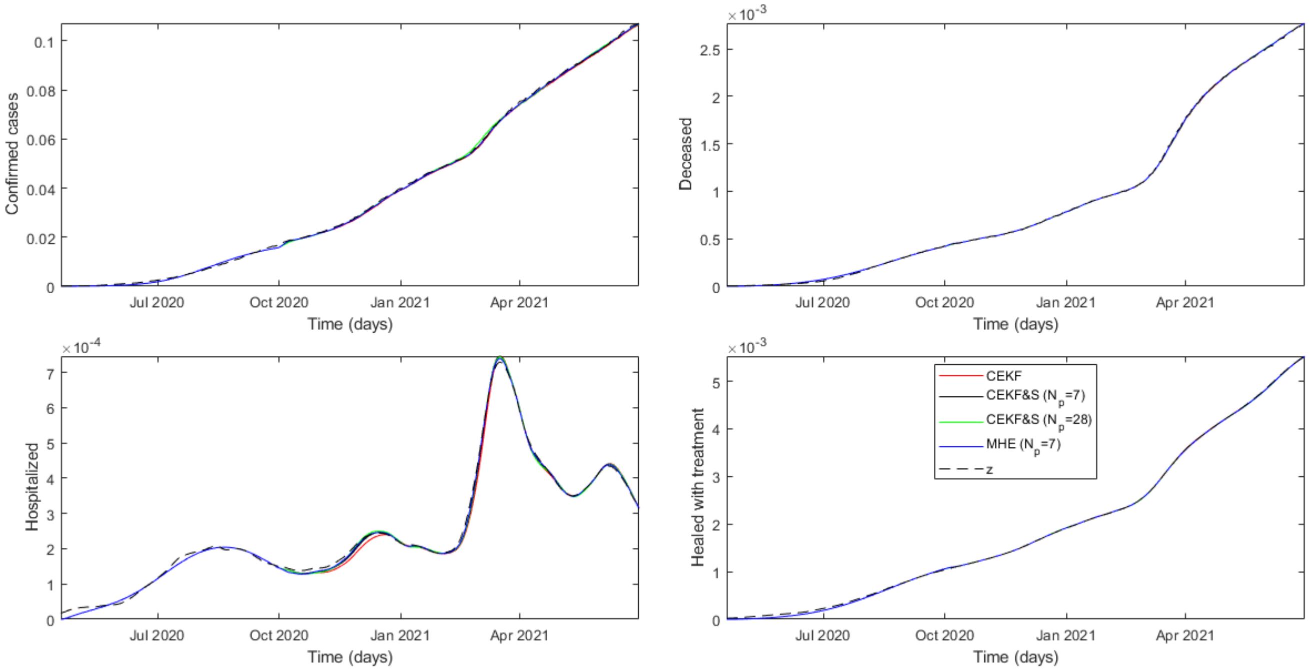

Figure S3. Time evolution of measures and estimated outputs from Rio Grande do Sul (RS).

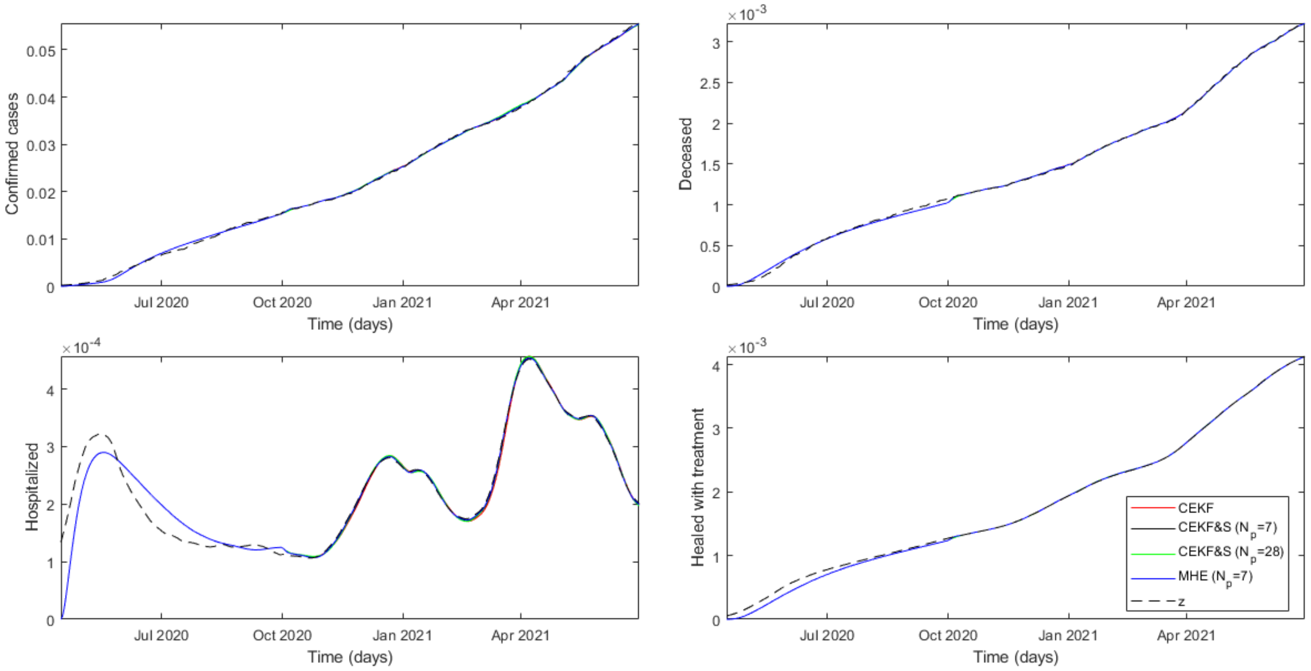

Figure S4. Time evolution of measures and estimated outputs from Rio de Janeiro (RJ).

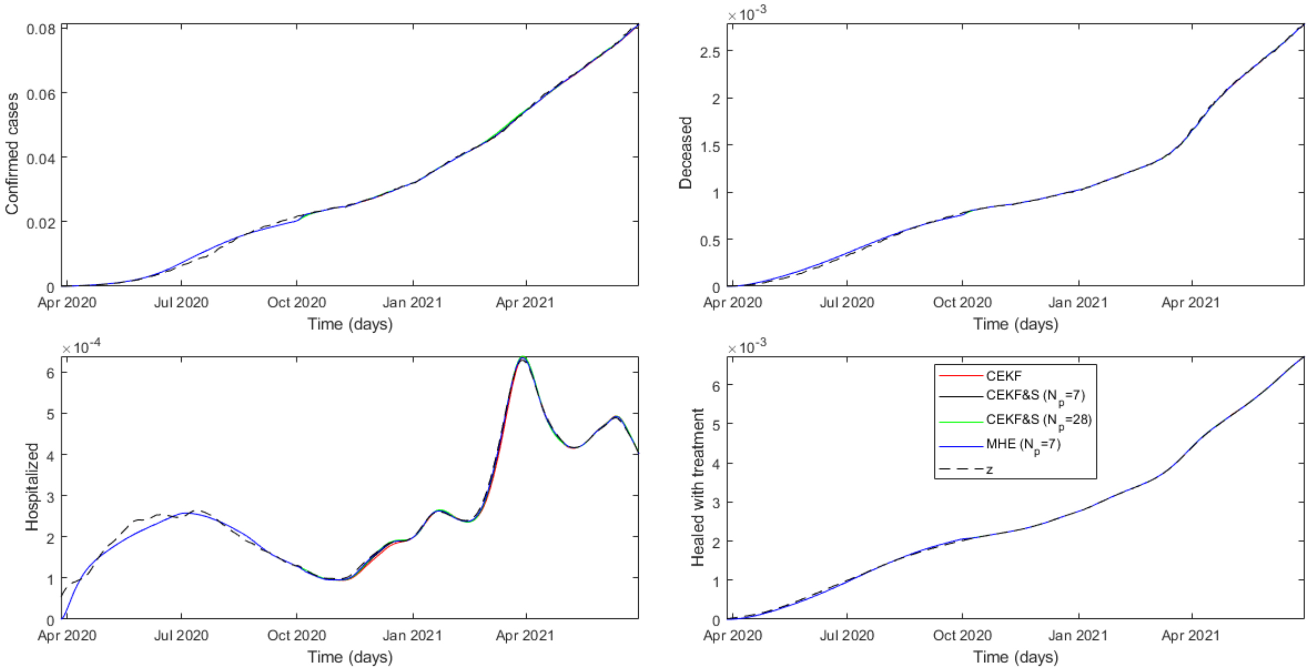

Figure S5. Time evolution of measures and estimated outputs from São Paulo (SP).
